# Mango wiggler as a novel insertion device providing a large and symmetrical imaging field of view

**DOI:** 10.1107/S1600577524004429

**Published:** 2024-06-21

**Authors:** Dongni Zhang, Ming Li, Xiaoyu Li, Huihua Lu, Yuhui Li, Jie Zhang, Gang Li, Weiwei Zhang, Yuhui Dong, Ye Tao, Weifan Sheng, Peng Liu

**Affiliations:** a Institute of High Energy Physics, Chinese Academy of Sciences 19B Yuquan Road Beijing100049 People’s Republic of China; b University of Chinese Academy of Sciences Yuquan Road Beijing100049 People’s Republic of China; University of Tokyo, Japan

**Keywords:** insertion device, wiggler, Mango wiggler, synchrotron radiation imaging, high energy photon source

## Abstract

A novel insertion device is introduced, designated as the Mango wiggler, designed for synchrotron radiation (SR) imaging that provides a large field of view. This innovative device is constructed from two orthogonal planar wigglers with a small difference in their period lengths, eliciting the phase difference of the magnetic fields to incrementally transitions from 0 to π/2. Such a configuration enlarges the vertical divergence of the light source, as with the horizontal divergence. The appellation ‘Mango wiggler’ derives from the distinctive mango-shaped contour of its radiation field. A comprehensive suite of theoretical analyses and simulations has been executed to elucidate the radiation properties of the Mango wiggler, employing *SPECTRA* and *Mathematica* as calculation tools. In conjunction with the ongoing construction of the High Energy Photon Source in Beijing a practical Mango wiggler device has been fabricated for utilization in SR imaging applications. Theoretical analyses were applied to this particular Mango wiggler to yield several theoretical conclusions, and several simulations were performed according to the measured magnetic field results.

## Abstract 2

A novel insertion device called the Mango wiggler has been proposed and developed for the High Energy Photon Source in Beijing, providing a large and symmetrical imaging field of view.

## 1. Introduction

**Figure 1:**
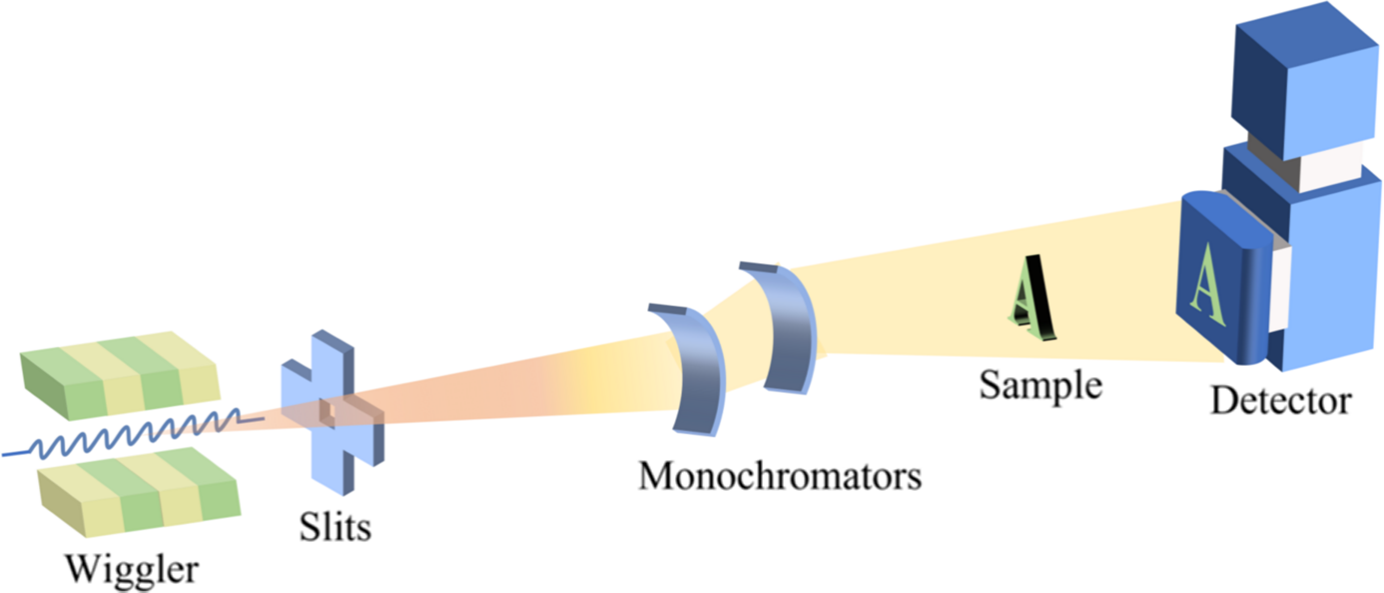
Typical schematic display of SR X-ray in-line imaging.

The construction of the High Energy Photon Source (HEPS) (Xiaoming *et al.*, 2014 ▸), a fourth-generation synchrotron radiation (SR) source, is currently underway. This advanced facility will feature a beamline dedicated to X-ray in-line imaging (Snigirev *et al.*, 1995 ▸) as demonstrated in Fig. 1 ▸. This beamline will support the *in situ*, multiscale, multicontrast and nondestructive study of the structure of advanced engineering materials and components. It will also support the precise, mesoscopic, nondestructive and high-sensitivity three-dimensional imaging of complete organs. Moreover, it will support the high-sensitivity, high-penetration and nondestructive three-dimensional imaging of palaeontology samples and human remains. The typical sample sizes involved in these studies vary greatly, as does the X-ray absorption; thus, the photon energy required for ideal SR X-ray in-line imaging also greatly varies. Many important studies, such as precise mesoscopic imaging of intact large organs (Landhuis, 2017 ▸), require a field of view (FOV) of the order of centimetres or even tens of centimetres and a spatial resolution in the micrometre or even submicrometre range. A large FOV with a high resolution is desirable for all imaging systems. Conventional planar wigglers, which are commonly used as X-ray sources for in-line imaging, have limitations in terms of their narrow radiation FOV due to the two-dimensional deflection of electrons within the wiggler. This results in a smaller vertical divergence compared with horizontal divergence, necessitating the scanning and stitching of images for large samples (Xing *et al.*, 2016 ▸), which can be inefficient and complex. From Fig. 1 ▸. the FOV of the in-line imaging depends on the divergence of the light source, and the spatial resolution depends on the source size observed at a point on the sample, which approximates the source size at a given angle. To address these issues, there is a clear need for a light source that can provide a larger divergence while maintaining a small enough source size to achieve real-time dynamic imaging with a large FOV.

To address the limitations of conventional planar wigglers and the need for a larger vertical divergence in SR imaging, various methods have been discussed in this article to modify the design of planar wigglers. These methods aim to expand the vertical divergence of the light source to achieve a larger FOV. One of the methods designated as the Mango wiggler with decisive advantages will be discussed in detail. The article commences with the genesis of the Mango wiggler, elucidating its conception. Subsequently, the radiation properties of the Mango wiggler are discussed, particularly its uneven angular distribution of radiation at both low and high energies. Both theoretical analyses and simulations are presented. The Mango wiggler device applied at HEPS is then presented. Theoretical analyses are applied to this practical Mango wiggler, and field measurement data of this actual device are used to perform some simulations. All the theoretical analyses and simulations are completed using *SPECTRA* (Tanaka, 2021 ▸) and *Mathematica* (Wolfram Research, 2018 ▸).

Two distinct Mango wigglers are utilized in the study. The first one is the ideal Mango wiggler (*B*_*x*0_ = *B*_*y*0_ = 1.8 T, λ_*ux*_ = 60.85 mm, λ_*uy*_ = 60 mm and *N*_*u*_ = 18), and the second one is the practical Mango wiggler at HEPS (*B*_*x*0_ = *B*_*y*0_ = 1 T, λ_*ux*_ = 50.70 mm, λ_*uy*_ = 50 mm, *N*_*ux*_ = 17.75 and *N*_*uy*_ = 18). The ideal Mango wiggler, which differs from the HEPS Mango wiggler in fundamental parameters, is used due to its stronger magnetic field. This allows it to generate more photons with higher energy, thus providing a clearer demonstration of radiation properties when using *SPECTRA*. The ideal Mango wiggler serves to illustrate the conceptual design and to derive theoretical analyses complemented by simulations in the discussion of radiation properties. These theoretical analyses are then applied to the HEPS Mango wiggler to yield theoretical outcomes. Additionally, several simulations are performed based on the measured magnetic field results of this practical Mango wiggler.

## 2. Concept of the Mango wiggler

Four methods to expand the vertical divergence of the light source are listed here. The first method is to add a bending magnet with a uniform horizontal field to a planar wiggler with a vertical field, abbreviated as the BP wiggler. The magnetic field strength of the added bending magnet should be much smaller than the peak field of the planar wiggler. Hence, the SR is mainly generated by the planar wiggler with a vertical field. Compared with the Mango wiggler presented in this section for which SR is produced by two wigglers in two directions, both the critical energy of the radiation and the flux generated in this first method are lower.

**Figure 2:**
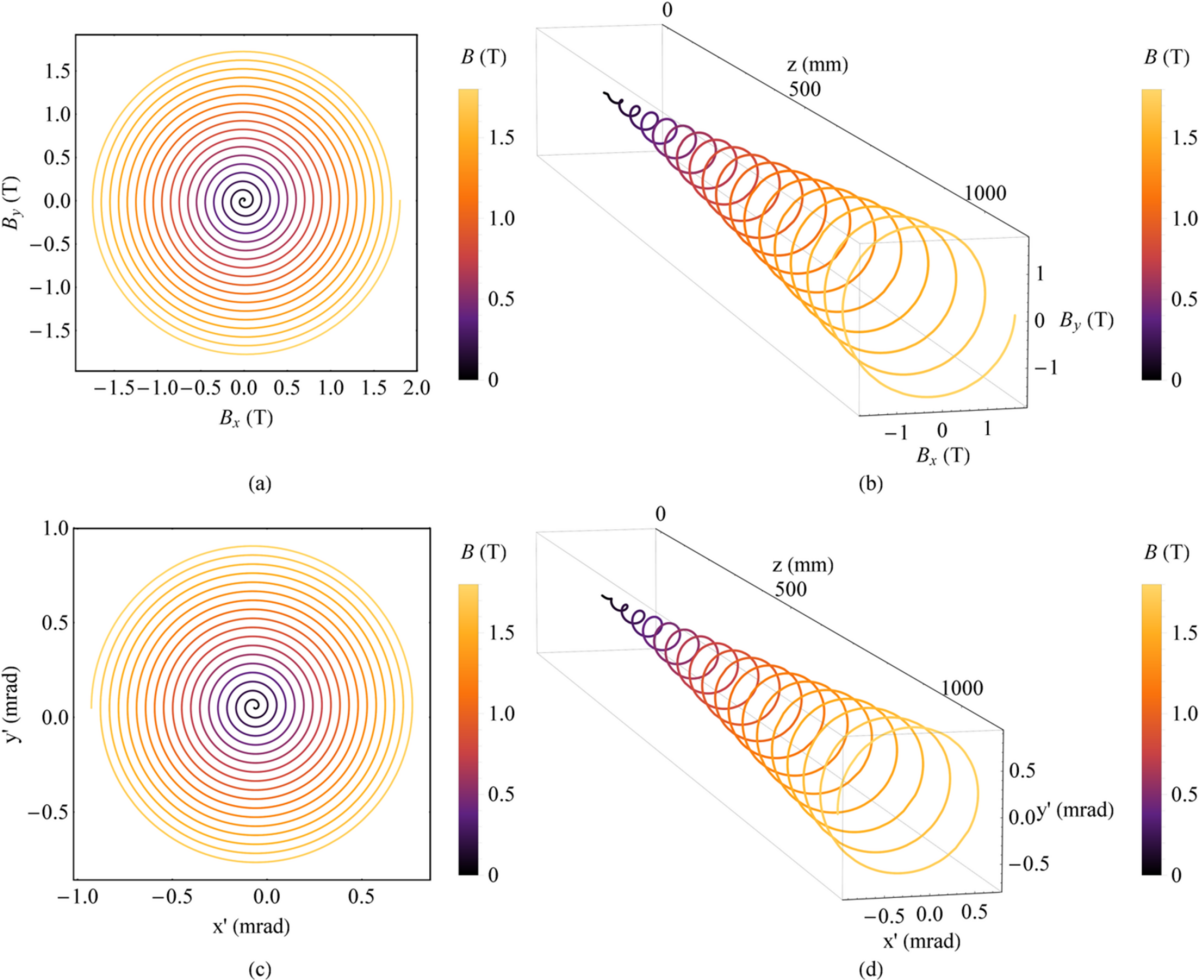
Magnetic field and angular trajectory of electrons in a spiral wiggler. (*a*) Magnetic field. (*b*) Magnetic field along *z*. (*c*) Angular trajectory of electrons. (*d*) Angular trajectory of electrons along *z*.

The second method is to use two orthogonal planar wigglers with the same peak magnetic field strength increasing from zero and a constant phase difference of π/2. The motion of electrons is gradually deflected from small circles to large circles along the beam axis. Thus, we call this a spiral wiggler. For instance, the magnetic fields of the two planar wigglers are defined as follows: *B*_*x*_(*z*) = *B*_*x*0_(*z*)sin(2π*z/λ_*ux*_* + π/2) and *B*_*y*_(*z*) = *B*_*y*0_(*z*)sin(2π*z/λ_*uy*_*). The peak magnetic field strengths *B*_*x*0_(*z*) and *B*_*y*0_(*z*) are the same, and both increase from 0 to 1.8 T. The period lengths *λ_*ux*_* and λ_*uy*_ are both 60 mm, and the number of periods *N*_*u*_ is 18. The magnetic field and the angular trajectory of electrons are plotted in Fig. 2 ▸.

**Figure 3:**
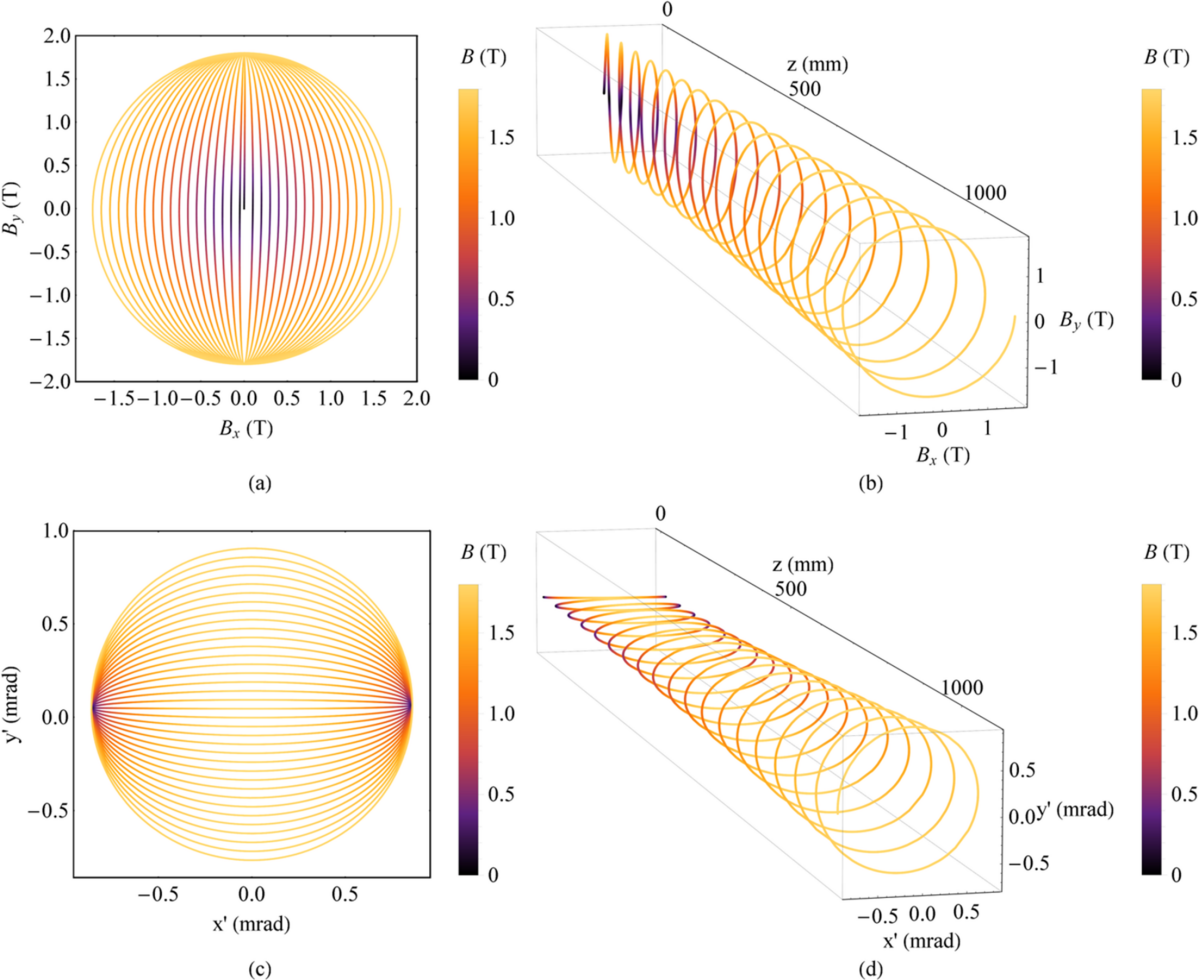
Magnetic field and angular trajectory of electrons in two combined wigglers with a constant phase difference of π/2 but different peak magnetic field strengths. (*a*) Magnetic field. (*b*) Magnetic field along *z*. (*c*) Angular trajectory of electrons. (*d*) Angular trajectory of electrons along *z*.

The third method is to use two orthogonal planar wigglers with a constant phase difference of π/2 but different peak magnetic field strengths. The horizontal peak magnetic field strength increases from zero while the vertical peak magnetic field strength is a constant. For instance, the magnetic fields for the two planar wigglers are *B*_*x*_(*z*) = *B*_*x*0_(*z*)sin(2π*z/λ_*ux*_* + π/2) and *B*_*y*_(*z*) = *B*_*y*0_sin(2π*z/λ_*uy*_*). *B*_*x*0_(*z*) increases from 0 to 1.8 T, and *B*_*y*0_ is 1.8 T. The period lengths *λ_*ux*_* and *λ_*uy*_* are both 60 mm, and the number of periods *N*_*u*_ is 18. The magnetic field and the angular trajectory of electrons are plotted in Fig. 3 ▸.

However, the magnetic field strength is too weak in the central area where *x*′ = 0 and *y*′ = 0 in Fig. 2 ▸(*c*). Therefore, both the power of the radiation and the flux of high-energy photons are very low due to the weak magnetic field strength in the centre of the FOV for the spiral wiggler. Moreover, realizing a magnetic field strength starting from a value of zero is very difficult in the design, processing, installing and adjusting.

**Figure 4:**
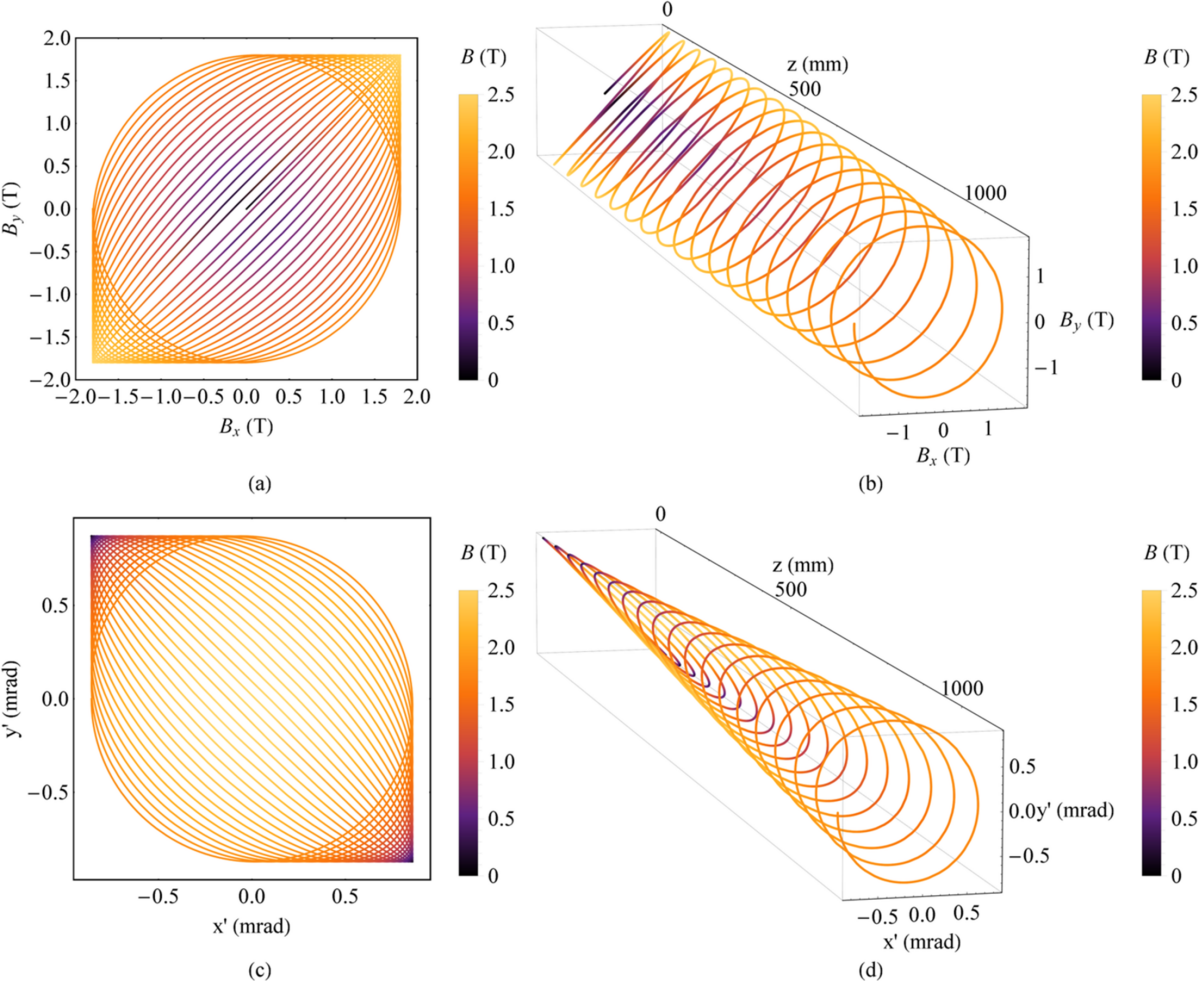
Magnetic field and angular trajectory of electrons in the ideal Mango wiggler. (*a*) Magnetic field. (*b*) Magnetic field along *z*. (*c*) Angular trajectory of electrons. (*d*) Angular trajectory of electrons along *z*.

The fourth method is still to use two orthogonal planar wigglers but for both directions the peak magnetic field strengths are the same constant value. There is a small difference in the period length for two directions, which causes the electron motion to vary from linear motion to circular motion. The magnetic fields of these combined wigglers are illustrated as equations (1) and (2),
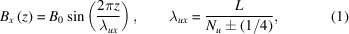

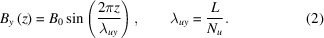
where *B*_0_ is the constant peak magnetic field strength and *L* is the length of the whole magnet. For instance, the peak magnetic field strength *B*_0_ is 1.8 T, the period length λ_*ux*_ is 60.85 mm, λ_*uy*_ is 60 mm and the number of periods *N*_*u*_ is 18 (Fig. 4 ▸). The strongest combined magnetic field strength is 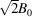. Angular obits are evenly distributed. So, most of the angular space (*x*′–*y*′) is evenly covered with longitude lines (demonstrated in Fig. 12 of Section 3.2). And the expanded FOV is centrally symmetrical.

The Mango wiggler has the following advantages. First, the Mango wiggler has a large integral radiation power. Second, the Mango wiggler has a high critical energy. Third, the high-energy SR of the Mango wiggler is produced at the centre of the FOV. Fourth, the Mango wiggler can not only enlarge the FOV but also maintain good imaging resolution which will be discussed in detail in Section 3.4. Fifth, the magnetic field of the Mango wiggler is feasible in the design, processing, installing and adjusting.

## 3. Radiation properties of the Mango wiggler

The Mango wiggler appears to be capable of providing a large and uniform FOV [Fig. 4 ▸(*c*)]. The radiation distribution is still uneven at low energy and high energy for different reasons. In this section, the properties of the radiation at low energy and high energy will be analysed and discussed. In addition, the photon flux, the critical energy and the source size will be discussed.

### 3.1. Uneven radiation distribution at low energy

For periodic wigglers, including planar wigglers and the Mango wiggler, the spectral distribution at the beam axis is discrete at low energy due to the undulator effect. Considering the coupling between the spectrum and deviation angle, the discretization of the spectrum at low energy leads to an uneven angular distribution of radiation.

**Figure 5:**
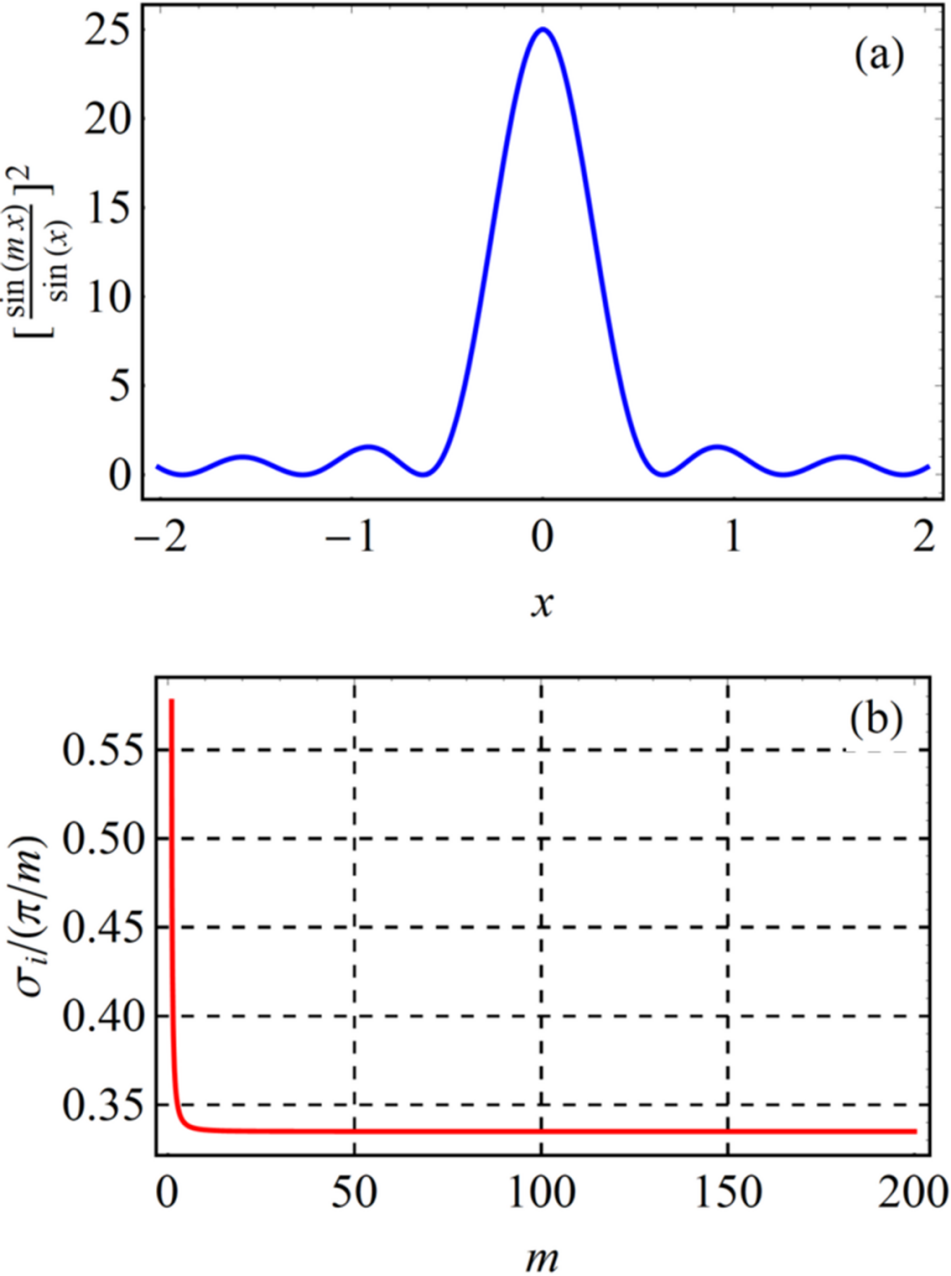
(*a*) The [sin(*mx*)/sin(*x*)]^2^ function when *m* = 5, for instance. (*b*) Ratio between the standard deviation and Rayleigh line width of [sin(*mx*)/sin(*x*)]^2^.

First, the SR of a single electron is considered. As derived from Kim (1989 ▸), the relative spectral line width for the *n*th harmonic is 1/(*nN*_*u*_). This line width is the distance between the main peak and closest valley according to the Rayleigh criterion (Rayleigh, 1879 ▸). In order to unify the width criterion during analysis, the standard deviation is used to describe the width. Therefore, the relative line width for the *n*th harmonic, 1/(*nN*_*u*_), needs to be transformed into a standard deviation form. The radiation mechanism from different magnetic poles is similar to multislit diffraction. The spectral distribution is regarded as a [sin(*mx*)/sin(*x*)]^2^-term function based on multislit diffraction theory, where *m* has a physical meaning of 2*N*_*u*_ for an insertion device. The Rayleigh line width of [sin(*mx*)/sin(*x*)]^2^ is π/*m* [Fig. 5 ▸*a*], and its standard deviation σ_*i*_ is defined in equation (3),
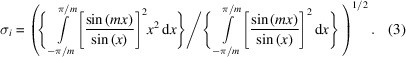
The ratio between the standard deviation and the Rayleigh line width is plotted in Fig. 5 ▸(*b*). The ratio is a constant value of 0.335 when *m* is large. Considering the reality of a wiggler, *m* is usually of the order of dozens. Thus, for the Rayleigh line width of the *n*th harmonic 1/(*nN*_*u*_), the corresponding standard deviation is 0.335/(*nN*_*u*_).

**Figure 6:**
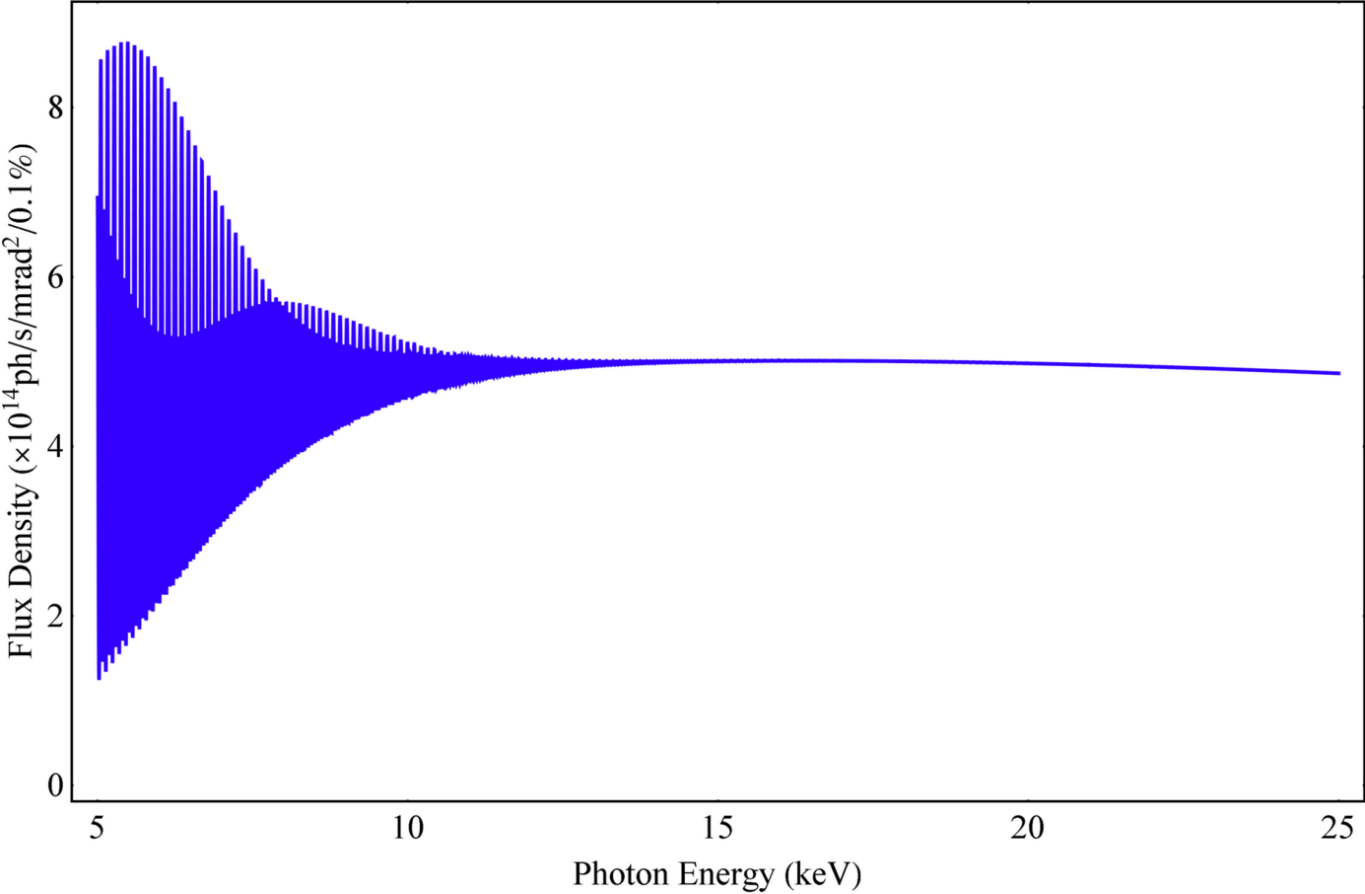
Flux density spectrum (5–30 keV) of the ideal Mango wiggler along the beam axis.

**Figure 8:**
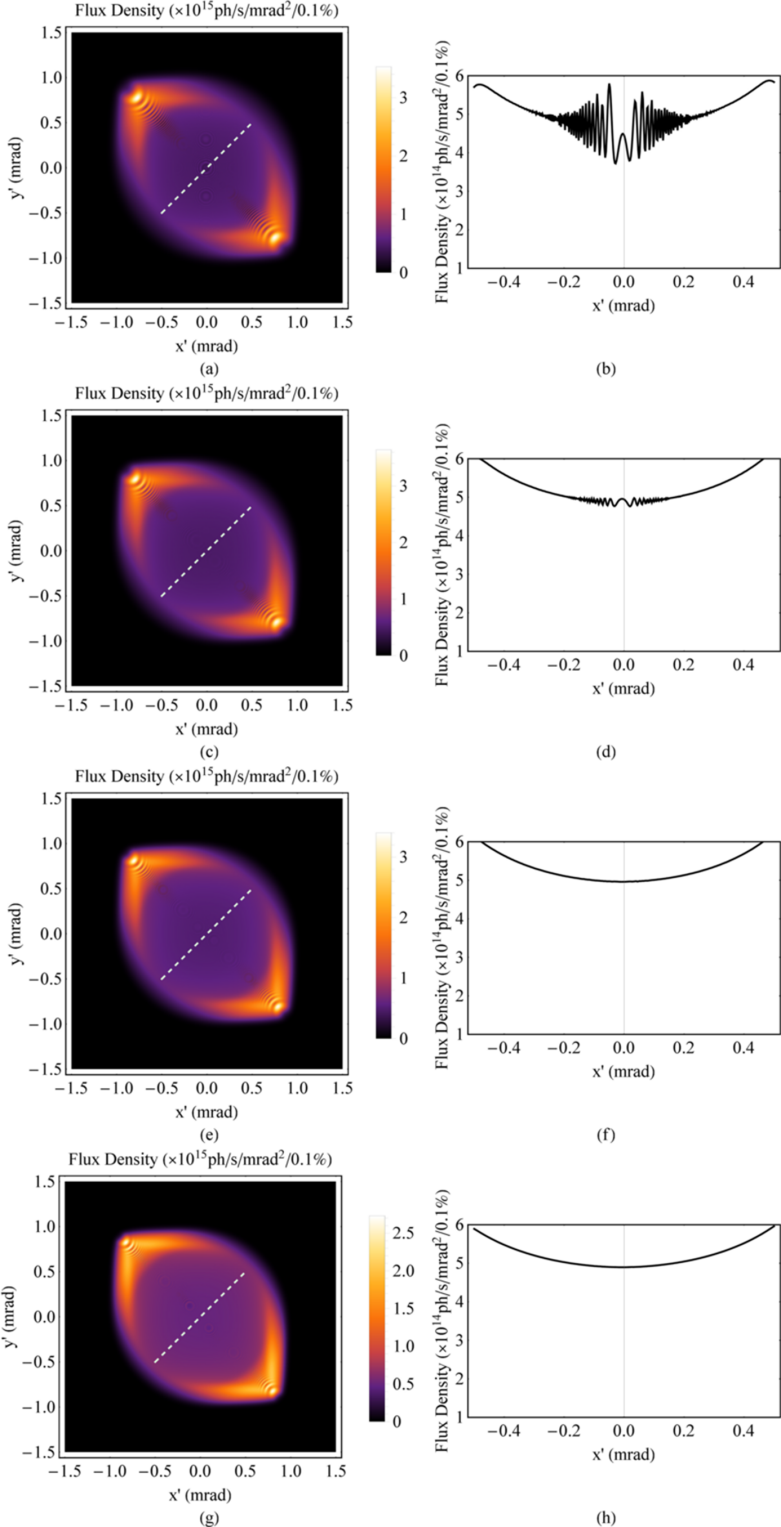
Angular distribution of the flux density at low energy of the ideal Mango wiggler. (*a*) SR energy at 8 keV, χ_1_ = 3.15. (*b*) One-dimensional distribution of the flux density along the 45° white dashed line in (*a*). (*c*) SR energy at 11.36 keV, χ_1_ = 2.22. (*d*) One-dimensional distribution of the flux density along the 45° white dashed line in (*c*). (*e*) SR energy at 16 keV, χ_1_ = 1.57. (*f*) One-dimensional distribution of the flux density along the 45° white dashed line in (*e*). (*g*) SR energy at 23 keV, χ_1_ = 1.10. (*h*) One-dimensional distribution of the flux density along the 45° white dashed line in (*g*).

**Figure 9:**
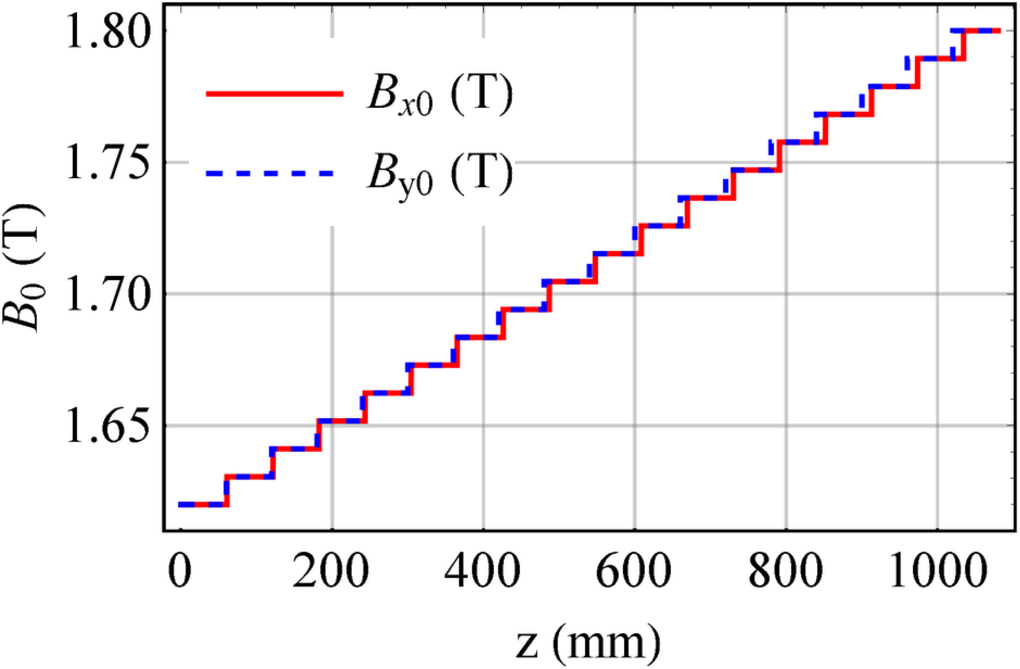
Magnetic fields of the tapered Mango wiggler.

**Figure 10:**
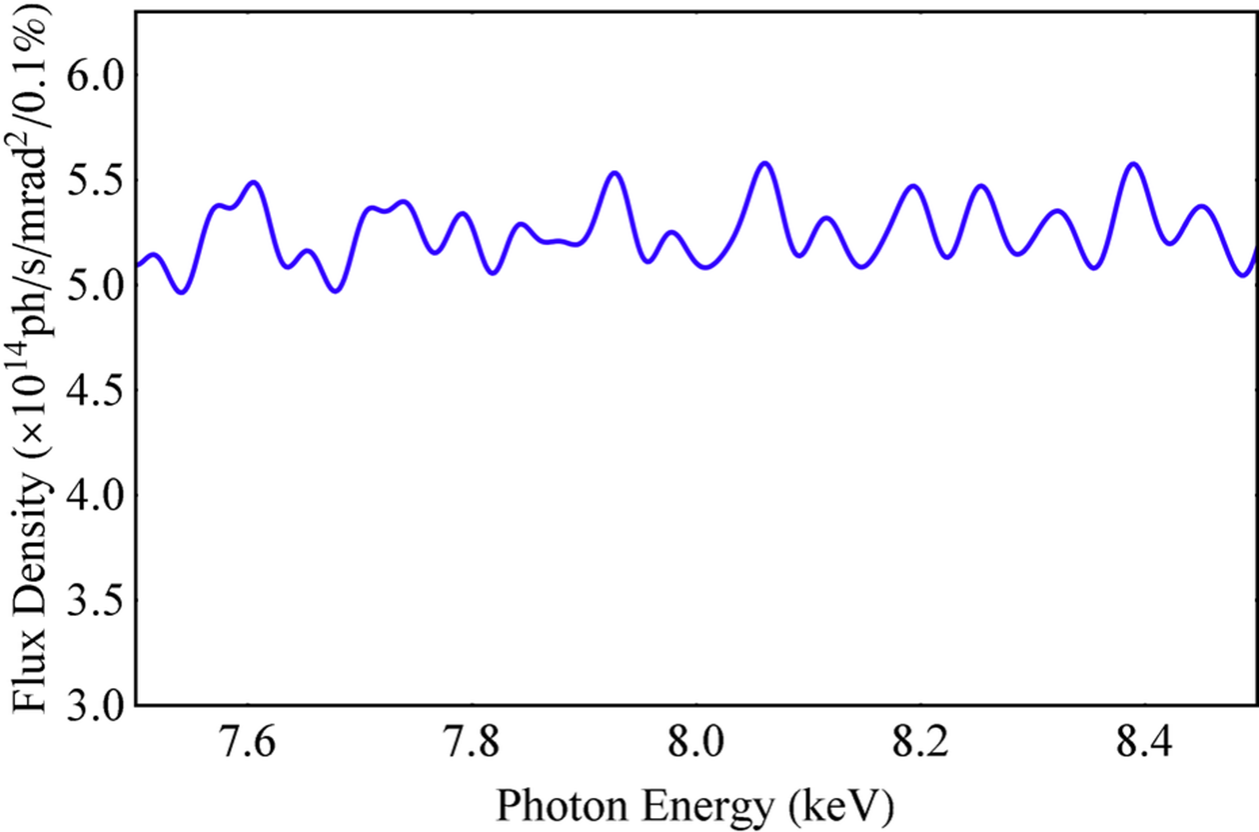
Flux density spectrum at 8 keV of the tapered Mango wiggler.

**Figure 11:**
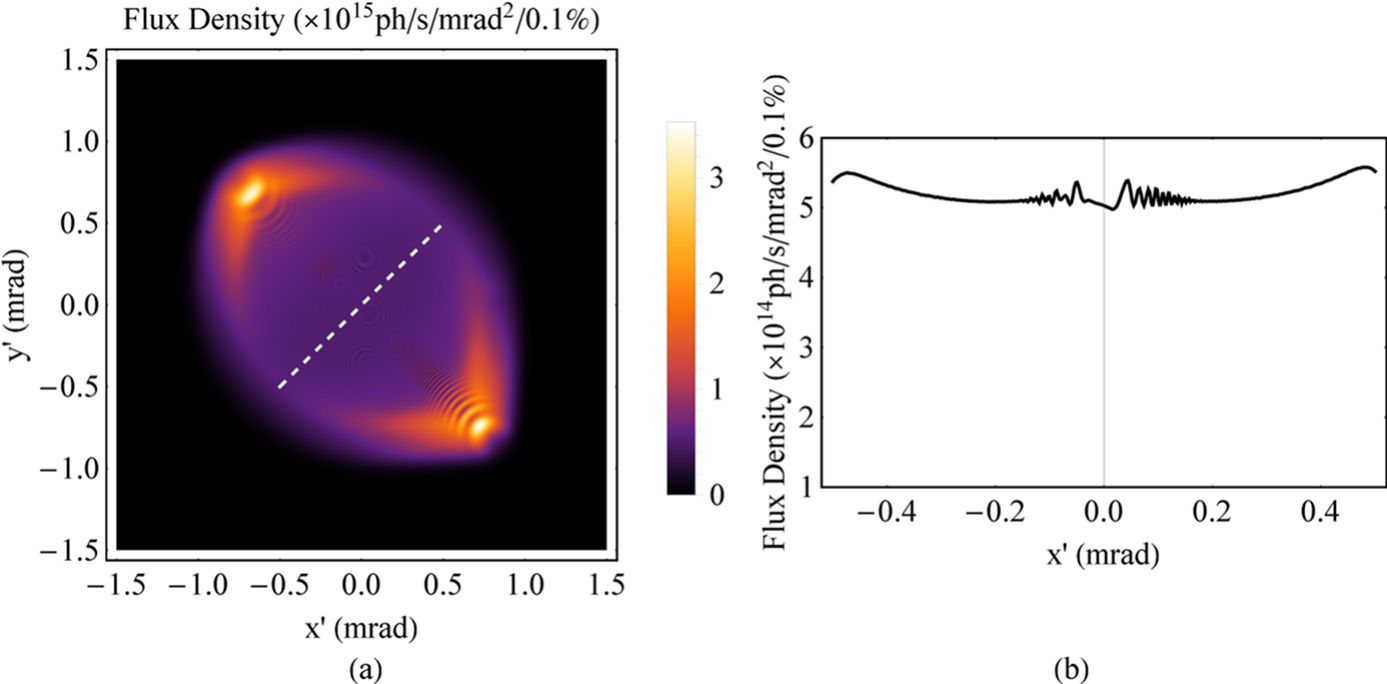
Angular distribution of the flux density at 8 keV of the tapered Mango wiggler. (*a*) Angular distribution of radiation. (*b*) One-dimensional distribution of the flux density along the 45° white dashed line in (*a*).

Second, the divergence of the electron bunch must be taken into account. The wavelength for the *n*th harmonic of a single electron observed at any deviation angle θ off the direction of the velocity of the single electron is illustrated as equation (4) (Kim, 1989 ▸), where *K* is the deflection parameter and γ is the particle energy in units of its rest mass. When observed along a single electron direction, where θ is zero, the wavelength for the *n*th harmonic is illustrated as equation (5). Therefore, the relative redshift for the *n*th harmonic of an electron with a deviation angle of θ when observed along the beam axis is illustrated as equation (6). The electrons distribution approximately follows a Gaussian distribution, and σ_θ_ is the standard deviation of the deviation angle θ of electrons in the electron bunch, with σ_θ_ = 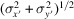, where σ_*x*′_ is known as the horizontal divergence of the electron bunch, and σ_*y*′_ is known as the vertical divergence of the electron bunch. Each deviating electron has a relative redshift for the *n*th harmonic when observed along the beam axis. The average relative redshift of electrons in the electron bunch is shown in equation (7). The degree of dispersion for all relative redshifts compared with the average relative redshift can be described by equation (8). For the Mango wiggler, the deflection parameter *K* is composed of deflection parameters from both the horizontal and vertical magnets, *K* = 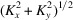, where *K*_*x*_ = *eB*_*x*0_λ_*ux*_/2π*m*_e_*c*, *K*_*y*_ = *eB*_*y*0_λ_*uy*_/2π*m*_e_*c*, *e* is the electron charge, *m*_e_ is the static mass of the electron and *c* is the velocity of light.
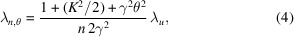

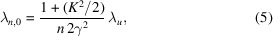

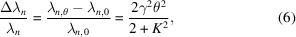

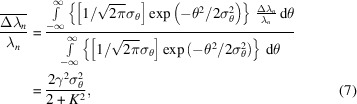

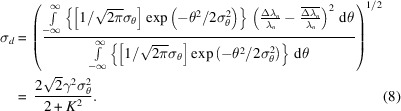
Third, the energy spread of the electron bunch δ_e_ is taken into account. When observed along the beam axis, the SR energy for the *n*th harmonic ɛ_*n*,0_ is illustrated as equation (9), where *E* is the electron energy (Kim, 1989 ▸). Then, equations (10) and (11) are derived from equation (9),
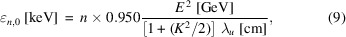

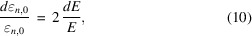

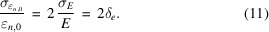
In summary, the spectral width δ_*n*,0_ for the *n*th harmonic along the beam axis is given by equation (12), ignoring the phase errors of the magnets here,
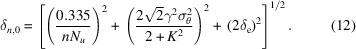
Whether the spectrum is uniformly distributed depends on the relationship between the spectral width and the distance between any two adjacent harmonics in the spectrum. When adjacent harmonics are much closer to each other, their spectral widths will overlap much more, and the spectrum is more uniform. The relative distance between any two adjacent harmonics in the spectrum is 1/*n*. A parameter is defined here to determine the uniformity of the spectrum, χ_1_, as presented in equation (13). The ideal Mango wiggler with the parameters in Section 2 is utilized to simulate the flux density with an energy dependence along the beam axis and an angular dependence via *SPECTRA* (Figs. 6 ▸, 7 ▸ and 8 ▸). The spectrum and angular distribution of radiation are both uniform for those harmonics for which χ_1_ is less than 1. The spectrum and angular distribution of radiation are uneven for those harmonics for which χ_1_ is greater than 3. 1 < χ_1_ < 3 is the transition region from uniformity to unevenness,
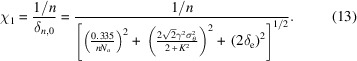
To make the discrete spectrum uniform so that the angular distribution of radiation is uniform, breaking up the magnetic periodicity is one approach, such as through tapering (Kroll *et al.*, 1981 ▸; Orzechowski *et al.*, 1986 ▸; Boyanov *et al.*, 1994 ▸). For instance, the magnetic fields of the ideal Mango wiggler presented in Section 2 can be tapered. *B*_*x*0_ and *B*_*y*0_ linearly increase from 1.62 T to 1.8 T (Fig. 9 ▸). Then, the spectrum and angular distribution of this tapered Mango wiggler at 8 keV are simulated (Figs. 10 ▸ and 11 ▸). The angular distribution of radiation at 8 keV becomes more uniform based on a comparison of Fig. 8 ▸(*b*) and Fig. 11 ▸(*b*).

### 3.2. Uneven distribution of radiation at high energy

**Figure 12:**
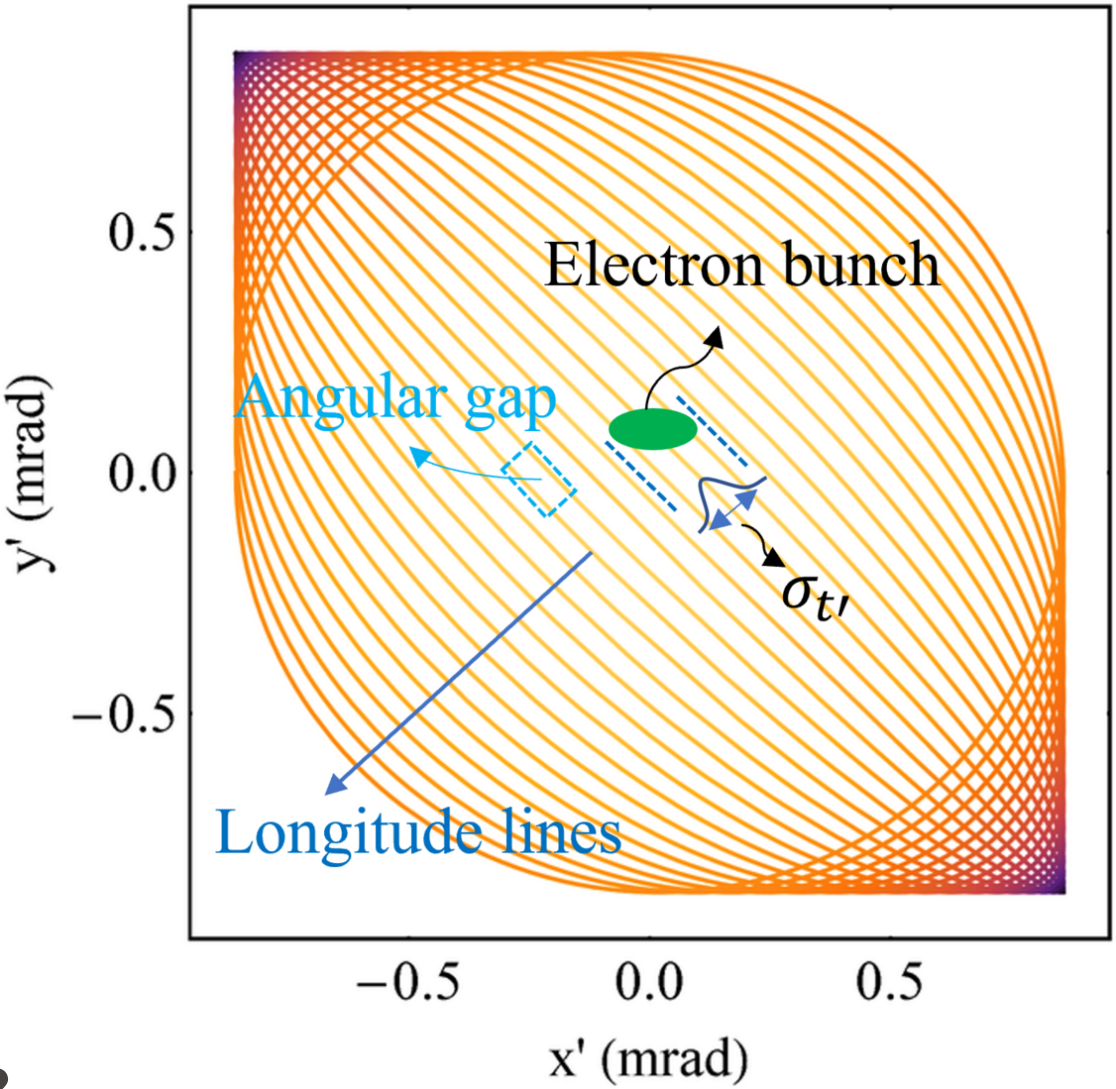
Demonstration of longitude lines, angular gaps among adjacent longitude lines, and divergence of the electron bunch in the 45° direction: σ_*t*′_.

As analysed above, the spectral harmonics can overlap with each other much more when the harmonics become higher for a planar wiggler. This property can ensure a uniform angular distribution of radiation for a planar wiggler at high energy but cannot guarantee that the Mango wiggler has a uniform angular distribution of radiation. In the radiation view of the Mango wiggler, the angular distribution of radiation produced by the divergence of the electron bunch and movement trajectory should be considered. Electrons in the Mango wiggler move in three-dimensional space rather than in a two-dimensional plane. There are angular gaps among the longitude lines in *x*′–*y*′ space (Fig. 12 ▸). The SR divergence decreases as the radiation energy increases. At high energy, the radiation divergence produced by the electron bunch is relatively too small for the gaps to form a uniform angular distribution of radiation.

The divergence of the SR produced by an electron bunch is the convolution between the SR produced by a single electron and the divergence of the electron bunch. The divergence of the SR produced by a single electron, σ_*r*′_, is illustrated in equation (14) (Kim, 1989 ▸),
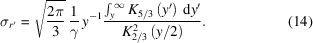
where *y* equals ε/ε_c_. ε_c_ is the critical energy of radiation, with ε_c_ = *h*ω_c_/2π. ω_c_ is the corresponding critical frequency, with *ω*_c_ = 3γ^3^*c*/2ρ. ρ is the radius of instantaneous curvature of the electron trajectory, with ρ = *m*_e_cγ/*eB*. *h* is the Planck constant. *K*_5/3_(*y*′) and *K*_2/3_(*y*/2) are modified Bessel functions. According to this equation, σ_*r*′_ decreases as the SR energy increases. The divergence of the electron bunch in the 45° direction is σ_*t*′_ = 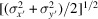
(Fig. 12 ▸), and the divergence of the SR produced by the electron bunch is σ_*T*′_ = 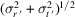. Therefore, σ_*T*′_ decreases as the SR energy increases.

**Figure 13:**
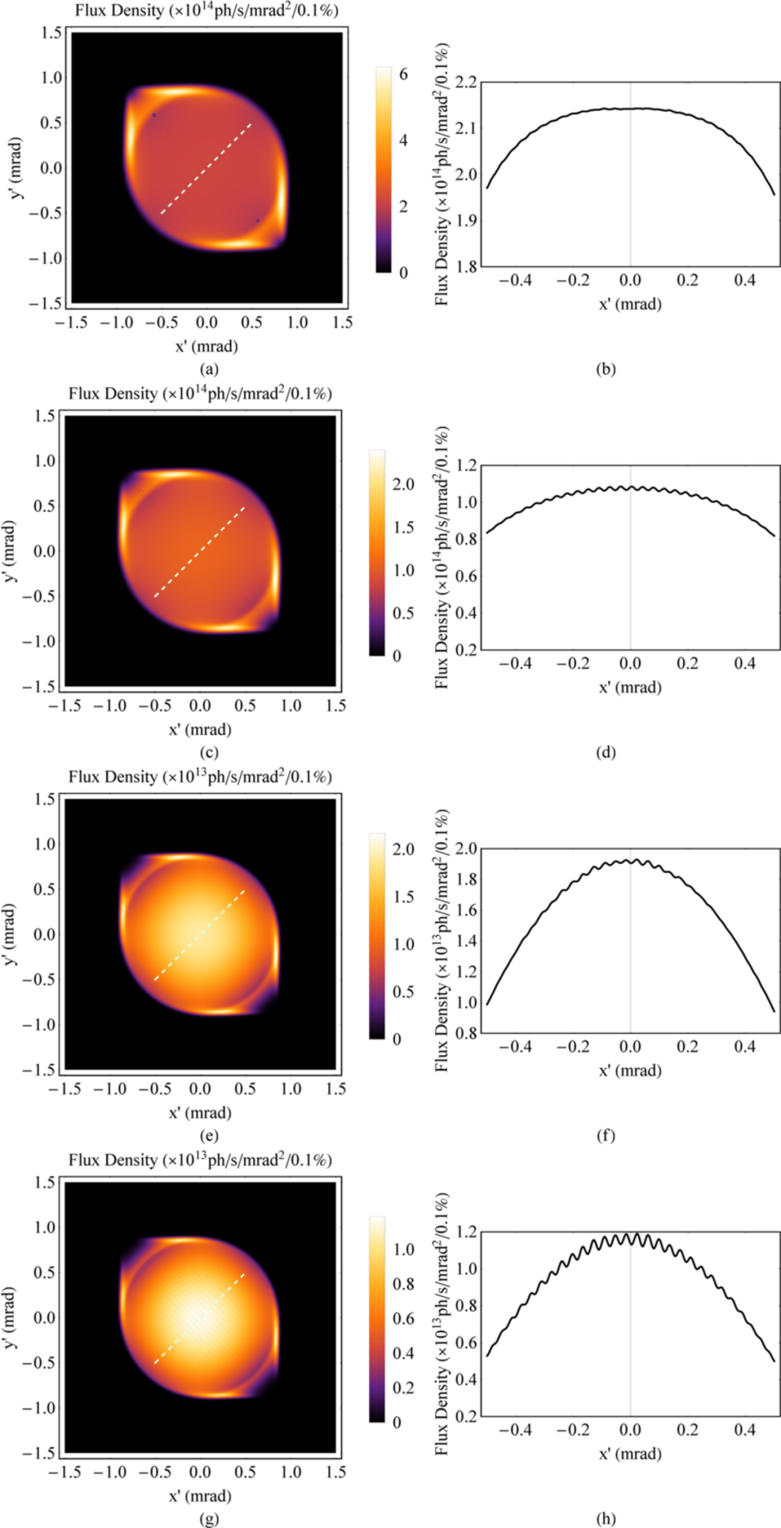
Angular distribution of the flux density at high energy of the ideal Mango wiggler. (*a*) SR energy at 100 keV, χ_2_ = 1.27. (*b*) One-dimensional distribution of the flux density along the 45° white dashed line in (*a*). (*c*) SR energy at 150 keV, χ_2_ = 1.58. (*d*) One-dimensional distribution of the flux density along the 45° white dashed line in (*c*). (*e*) SR energy at 267 keV, χ_2_ = 2.15. (*f*) One-dimensional distribution of the flux density along the 45° white dashed line in (*e*). (*g*) SR energy at 300 keV, χ_2_ = 2.29. (*h*) One-dimensional distribution of the flux density along the 45° white dashed line in (*g*).

The central angular gap *A*_g0_ is considered representative because it is the largest among all the gaps. *A*_g_ is used to describe the gap between any adjacent longitude lines, and *A*_g0_ is a subset of it. Since the period lengths in the two directions differ little, the phase difference of oscillation for electrons in the two directions Δφ after undergoing an average period of |*λ_*ux*_* − *λ_*uy*_*|/[(*λ_*ux*_* + λ_*uy*_)/2] is illustrated as equation (15). The angular motion formulas in the two directions are *x*′ =*K*_*x*_/γcosφ and *y*′ = *K*_*y*_/γcos(φ + Δφ), which are parametric functions. Thus, the central angular gap *A*_g0_ is derived based on analytical geometry and illustrated as equation (16). Whether the angular distribution of radiation at high energy is uniform depends on the relationship between *A*_g0_ and σ_*T*′_. Similar to the analysis at low energy, a parameter χ_2_ is defined here, which is illustrated in equation (17), to determine the uniformity of the angular distribution of radiation at high energy. From the simulation results of the ideal Mango wiggler, the following conclusions can be drawn. The angular distribution of radiation can be uniform for those energies with χ_2_ < 1, the angular distribution of radiation can be uneven for those energies with χ_2_ > 3, and 1 < χ_2_ < 3 is the transition region from a uniform angular distribution of radiation to an uneven angular distribution of radiation. Four simulation examples of the ideal Mango wiggler at high energy are shown in Fig. 13 ▸. Therefore, factors influencing uniformity at high energy can be analysed from equations (16) and (17). If SR with higher critical energy is required, the magnetic field strength *B*_*x*0_ and *B*_*y*0_ must be stronger. Then this will result in a larger *A*_g0_ in equation (16) as *B*_*x*0_ and *B*_*y*0_ become larger. SR with higher energy will result in a smaller σ_*T*′_ as mentioned before. Thus, χ_2_ becomes larger in equation (17). Considering this situation, in order to decrease χ_2_, the period length needs to be shortened. The challenge is the realization of strong magnetic fields with short periods length.
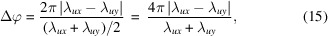

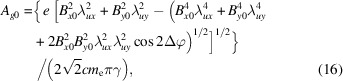

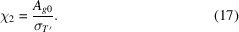


### 3.3. Photon flux and critical energy

**Figure 14:**
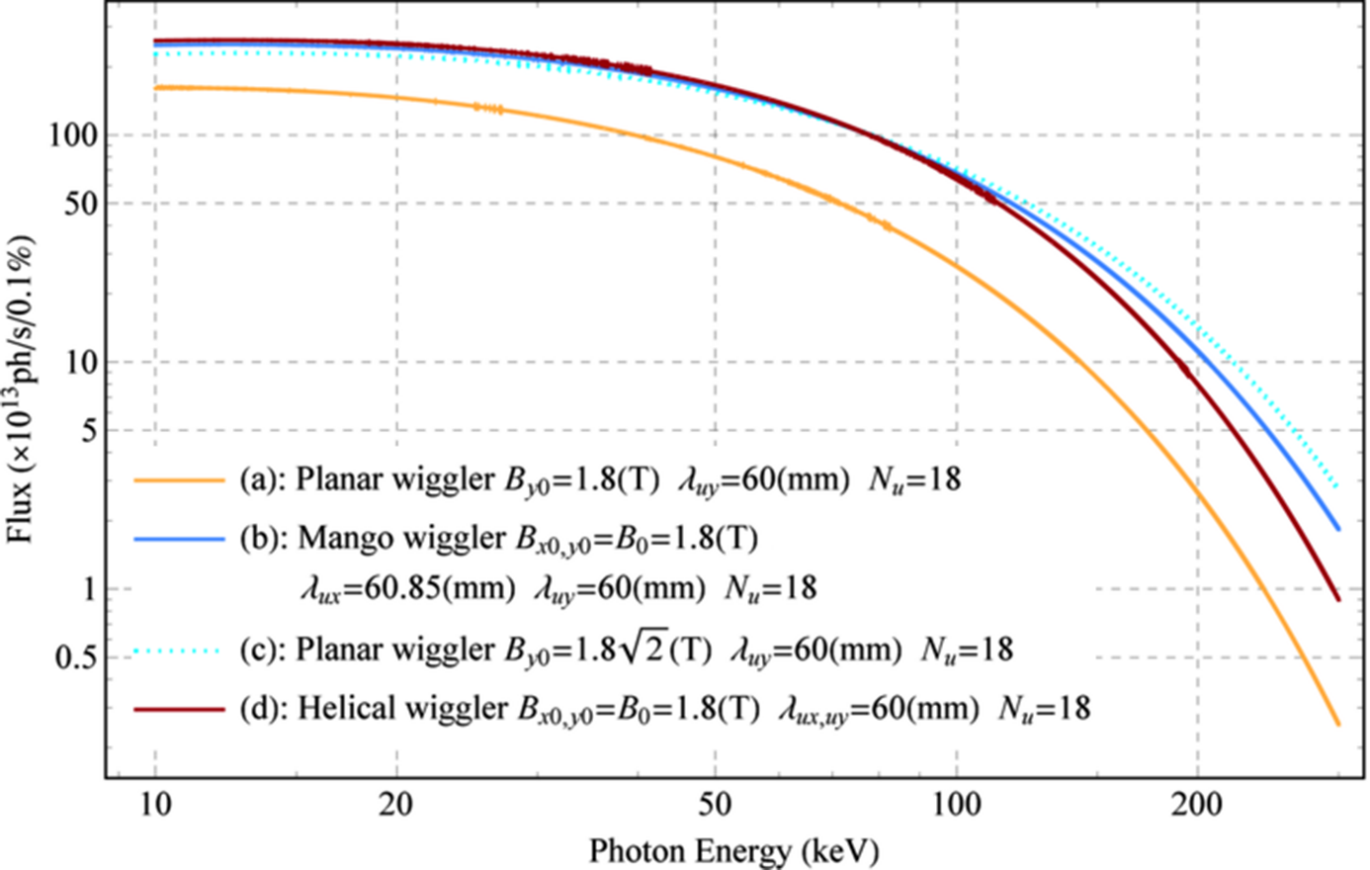
Photon fluxes within a 3 mrad acceptance angle for different wigglers. (*a*) Planar wiggler [*B*_*y*_(*z*) = *B*_*y*0_sin(2π*z*/λ_*uy*_), *B*_*y*0_ = 1.8 T, λ_*uy*_ = 60 mm and *N*_*u*_ = 18]. (*b*) The ideal Mango wiggler. (*c*) Planar wiggler [*B*_*y*_(*z*) = *B*_*y*0_sin(2π*z*/λ_*uy*_), *B*_*y*0_ = 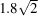
 T, λ_*uy*_ = 60 mm and *N*_*u*_ = 18]. (*d*) Helical wiggler [*B*_*x*_(*z*) = *B*_*x*0_cos(2π*z*/λ_*ux*_), *B*_*y*_(*z*) = *B*_*y*0_sin(2π*z*/λ_*uy*_), *B*_*x*0_ = *B*_*y*0_ = 1.8 T, λ_*ux*_ = λ_*uy*_ = 60 mm and *N*_*u*_ = 18].

**Figure 15:**
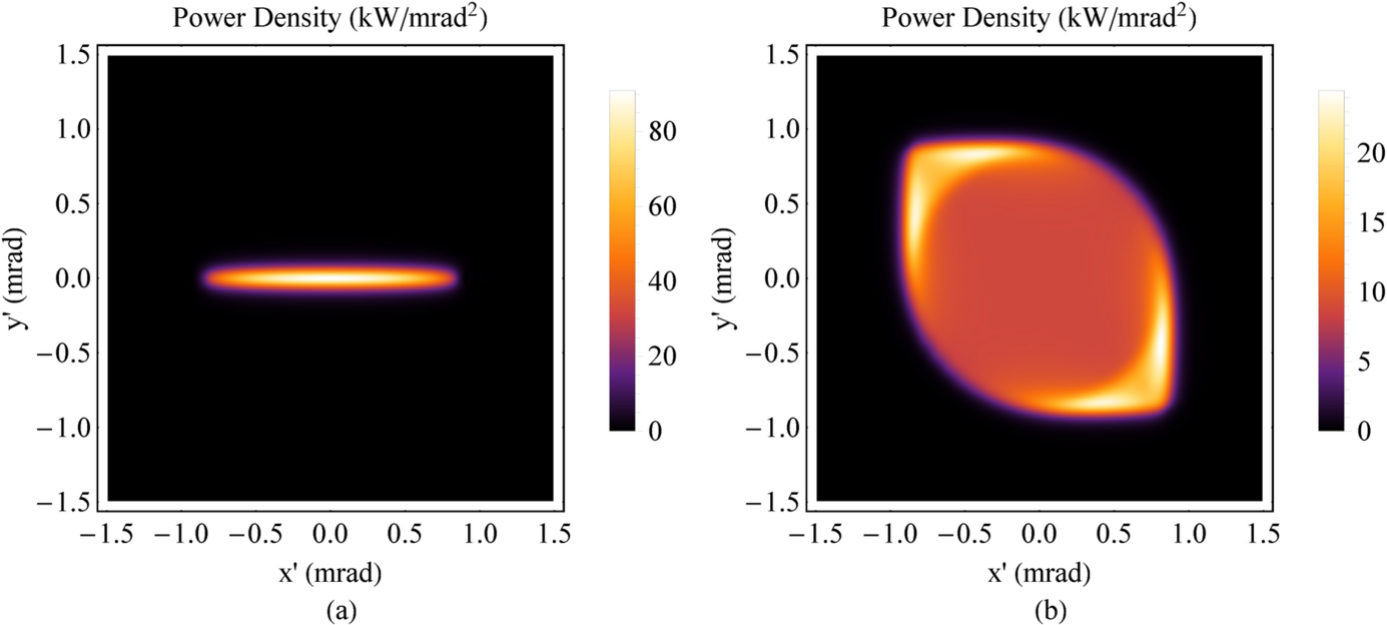
Angular distribution of the power density. (*a*) Planar wiggler [*B*_*y*_(*z*) = *B*_*y*0_sin(2π*z*/λ_*uy*_), *B*_*y*0_ = 1.8 T, λ_*uy*_ = 60 mm and *N*_*u*_ = 18]. (*b*) The ideal Mango wiggler.

The simulated photon fluxes of the ideal Mango wiggler and a planar wiggler [*B*_*y*_(*z*) = *B*_*y*0_sin(2π*z*/λ_*uy*_), *B*_*y*0_ = 1.8 T, *λ_*uy*_* = 60 mm and *N*_*u*_ = 18] are shown in Fig. 14 ▸ [curves (*b*) and (*a*)]. Another planar wiggler [*B*_*y*_(*z*) = *B*_*y*0_sin(2π*z*/λ_*uy*_), *B*_*y*0_ = 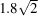
 T, λ_*uy*_ = 60 mm and *N*_*u*_ = 18] [Fig. 14 ▸, curve (*c*)] and a helical wiggler [the magnetic field resembles a helical undulator (Alferov *et al.*, 1974 ▸)] [*B*_*x*_(*z*) = *B*_*x*0_cos(2π*z*/λ_*ux*_), *B*_*y*_(*z*) = *B*_*y*0_sin(2π*z*/λ_*uy*_), *B*_*x*0_ = *B*_*y*0_ = 1.8 T, λ_*ux*_ = *λ_*uy*_* = 60 mm and *N*_*u*_ = 18] [Fig. 14 ▸, curve (*d*)] are also simulated. The critical energy of the planar wiggler is 3*B*_0_*h*γ^2^*e*/4π*m*_e_ (Thompson & Vaughan, 2001 ▸). The critical energy of the Mango wiggler changes gradually from 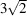
*B*_0_*h*γ^2^*e*/4π*m*_e_ to 3*B*_0_*h*γ^2^*e*/4π*m*_e_ along *z*, and from Fig. 4 ▸(*c*) the higher critical energy appears at the central area of SR angular distribution. From the comparison, the Mango wiggler provides not only a larger FOV (see Fig. 15 ▸, angular distribution of the power density) but also a higher photon flux and a higher critical energy than the planar wiggler, as shown in Fig. 14 ▸ [curve (*a*)]. Moreover, the flux of the Mango wiggler is between those of the planar wiggler with a higher magnetic field, shown in Fig. 14 ▸ [curve (*c*)], and the helical wiggler, shown in Fig. 14 ▸ [curve (*d*)].

### 3.4. The source size at a given angle

**Figure 16:**
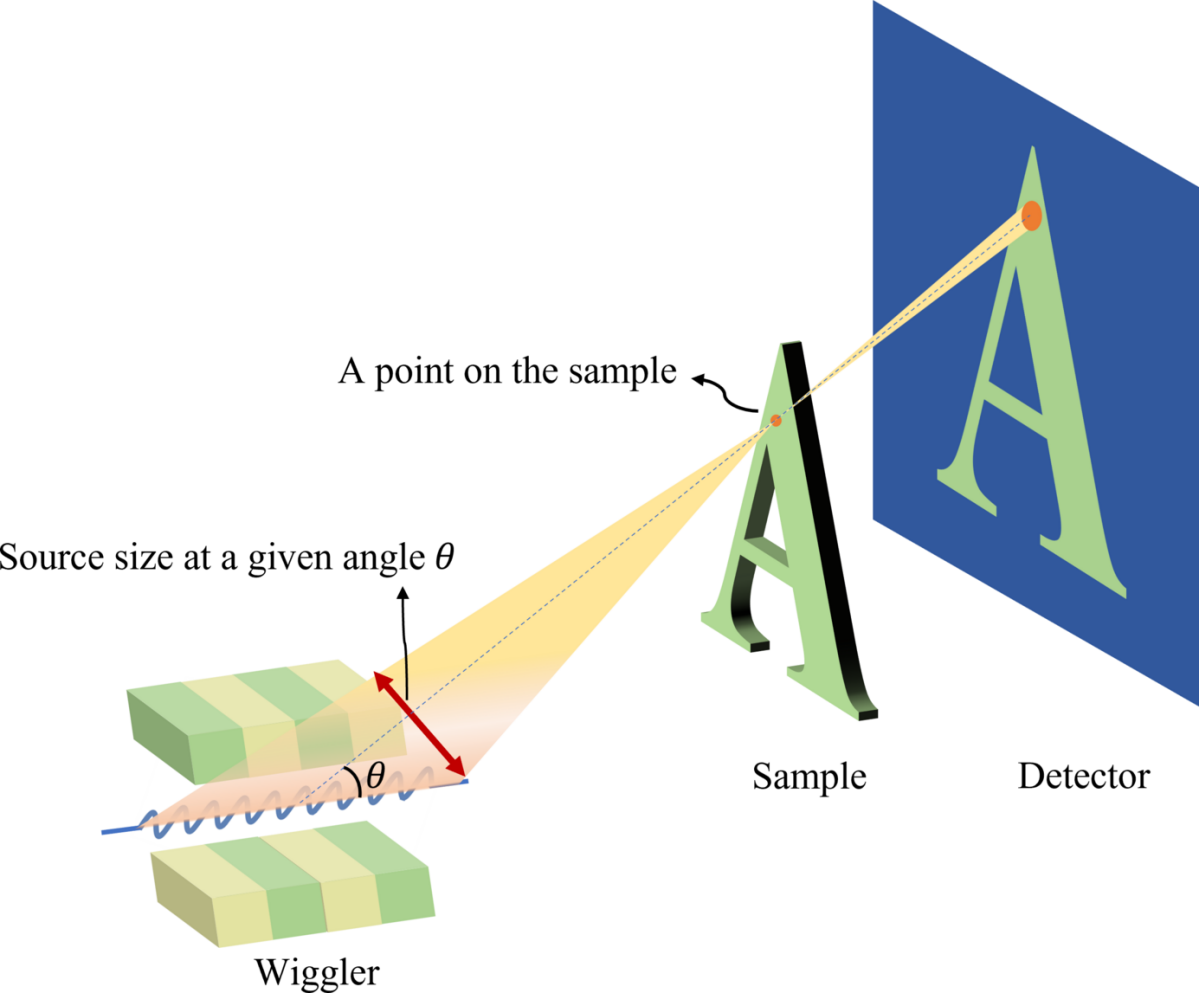
The source size at the end of wiggler observed from a point in the sample.

For the applications of X-ray in-line imaging, the source size at a given angle influencing imaging resolution refers to the source size at the end of the wiggler observed from a point in the sample, shown in Fig. 16 ▸. For a planar wiggler, the source size at a given angle θ_*x*_ has been derived from Walker (2003 ▸, 2017 ▸) and illustrated as equation (18). The transverse size of the electron bunch, the transverse oscillation and the oblique observation angle contribute to the source size at a given angle. Σ_*x*_ is the r.m.s. of the distribution at a given angle θ_*x*_, σ_*x*_ is the size of the electron bunch, σ_*x*′_ is the divergence of the electron bunch, *a* is the amplitude of sinusoidal oscillation of the motion of the electron bunch, and *L* is the length of the wiggler. For a Mango wiggler, the magnet length contributing to the formation of radiation emitted at a given angle is much shorter than the total wiggler length *L*. Therefore, 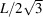 in the third and the fourth terms on the right side of equation (18) needs to be adjusted to λ_*u*_σ_*T*′_/*A*_g_ where λ_*u*_ = (*λ_*ux*_* + λ_*uy*_)/2 is the average period length. The source size of a Mango wiggler at a given angle (*x*′, *y*′) is illustrated in equation (19), only around −*x*′ = *y*′, *a*_*x*_ = *a*ζ_*x*_, *a*_*y*_ = *a*ζ_*y*_, *a* = δλ_*u*_/2π, δ = *K*/γ. ζ_*x*_ and ζ_*y*_ are illustrated as equations (20) and (21) separately. When |*x*′+ *y*′| is much larger than σ_*T*′_, *a*_*x*_ and *a*_*y*_ are both zero. Comparing equations (18) and (19), the source size at a given angle of the Mango wiggler is smaller than that of the planar wiggler. Thus, the Mango wiggler has more advantages in imaging resolution,
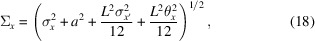

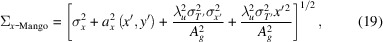

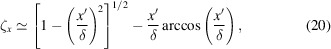

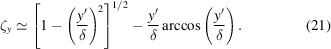


### 3.5. Some comparisons with the BP wiggler

**Figure 17:**
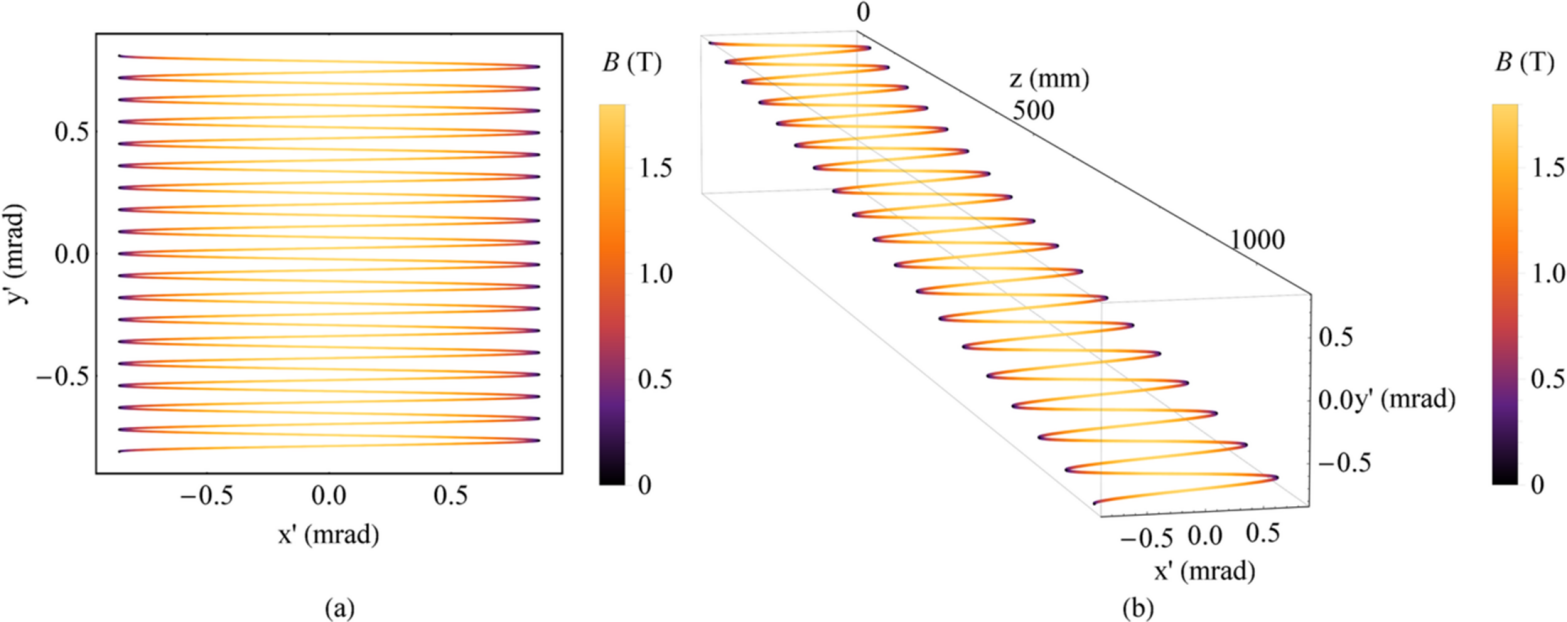
The angular trajectory of the BP wiggler: *B*_*x*0_ = 0.03 T, *B*_*y*_(*z*) = *B*_*y*0_sin(2π*z*/λ_*uy*_), *B*_*y*0_ = 1.8 T, λ_*uy*_ = 60 mm and *N*_*u*_ = 18.

The BP wiggler has the same effect of expanding FOV. Fig. 17 ▸ shows the angular trajectory of the BP wiggler [*B*_*x*0_ = 0.03 T, *B*_*y*_(*z*) = *B*_*y*0_sin(2π*z*/λ_*uy*_), *B*_*y*0_ = 1.8 T, λ_*uy*_ = 60 mm and*N*_*u*_ = 18].

**Figure 18:**
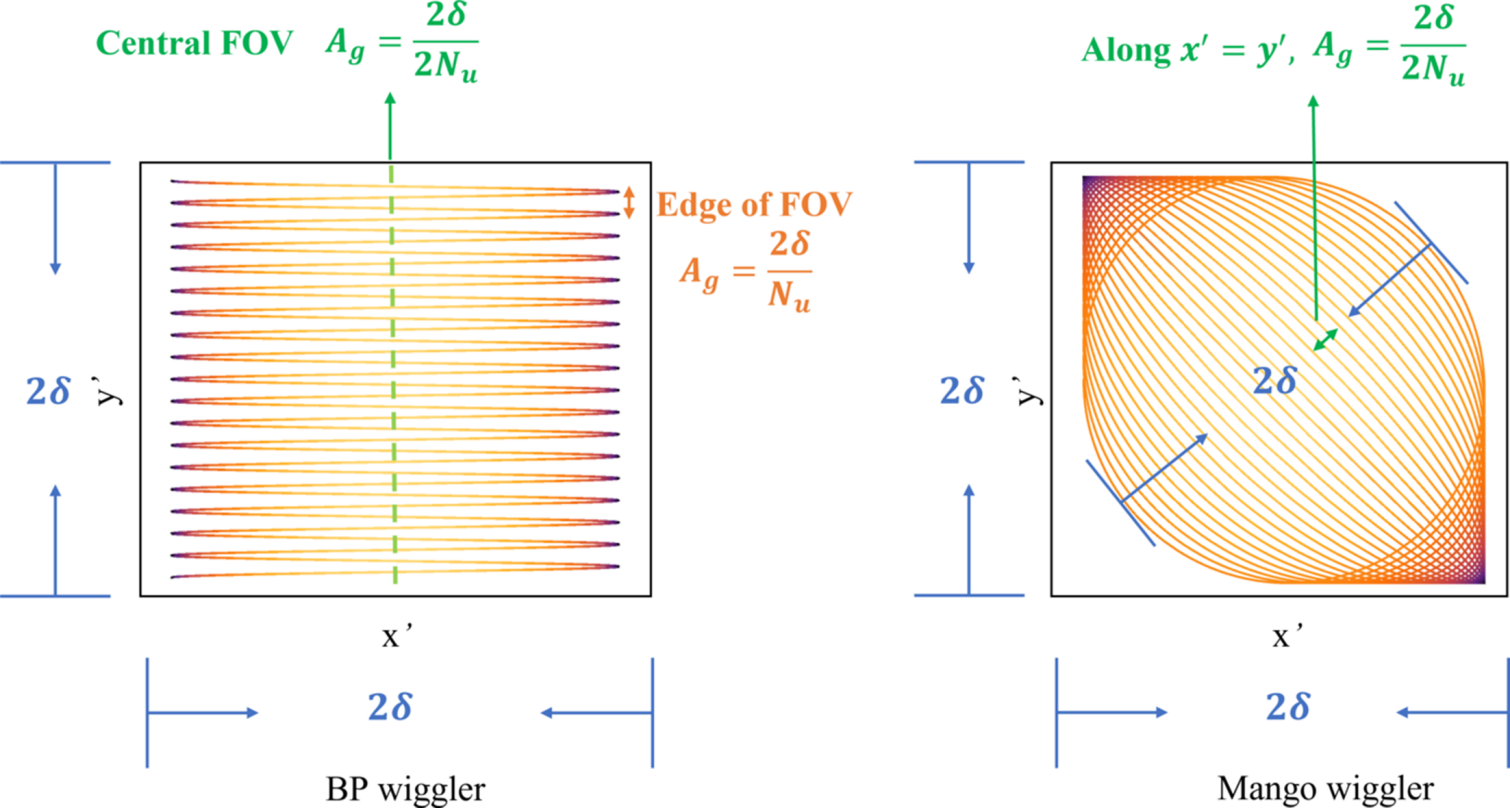
A comparison of the uniformity of the FOV between the BP wiggler and Mango wiggler.

The Mango wiggler has advantages in the uniformity of the FOV compared with the BP wiggler. If the vertical divergence is the same as that in the horizontal direction, which is 2δ, the angular gap in the central FOV of the BP wiggler will be 2δ/2*N*_*u*_ and the angular gap at the edges will be 2δ/*N*_*u*_ (Fig. 18 ▸). Even if the central FOV is uniform, it does not guarantee uniformity across the entire FOV. As for the Mango wiggler, since its trajectory of the electron bunch unfolds uniformly in space, the angular gap along *x*′ = *y*′ is the same, which is 2δ/2*N*_*u*_ (Fig. 18 ▸). If the central FOV is uniform, then the entire FOV must also be uniform.

The Mango wiggler has another advantage over the BP wiggler. Due to the vertical deflection in the BP wiggler, the orbit excursion gives rise to lifetime degradation (due to effective loss of the vertical physical aperture). The Mango wiggler can avoid such an issue and thus the minimum gap can be narrower than the BP wiggler.

## 4. The Mango wiggler at the HEPS

**Figure 19:**
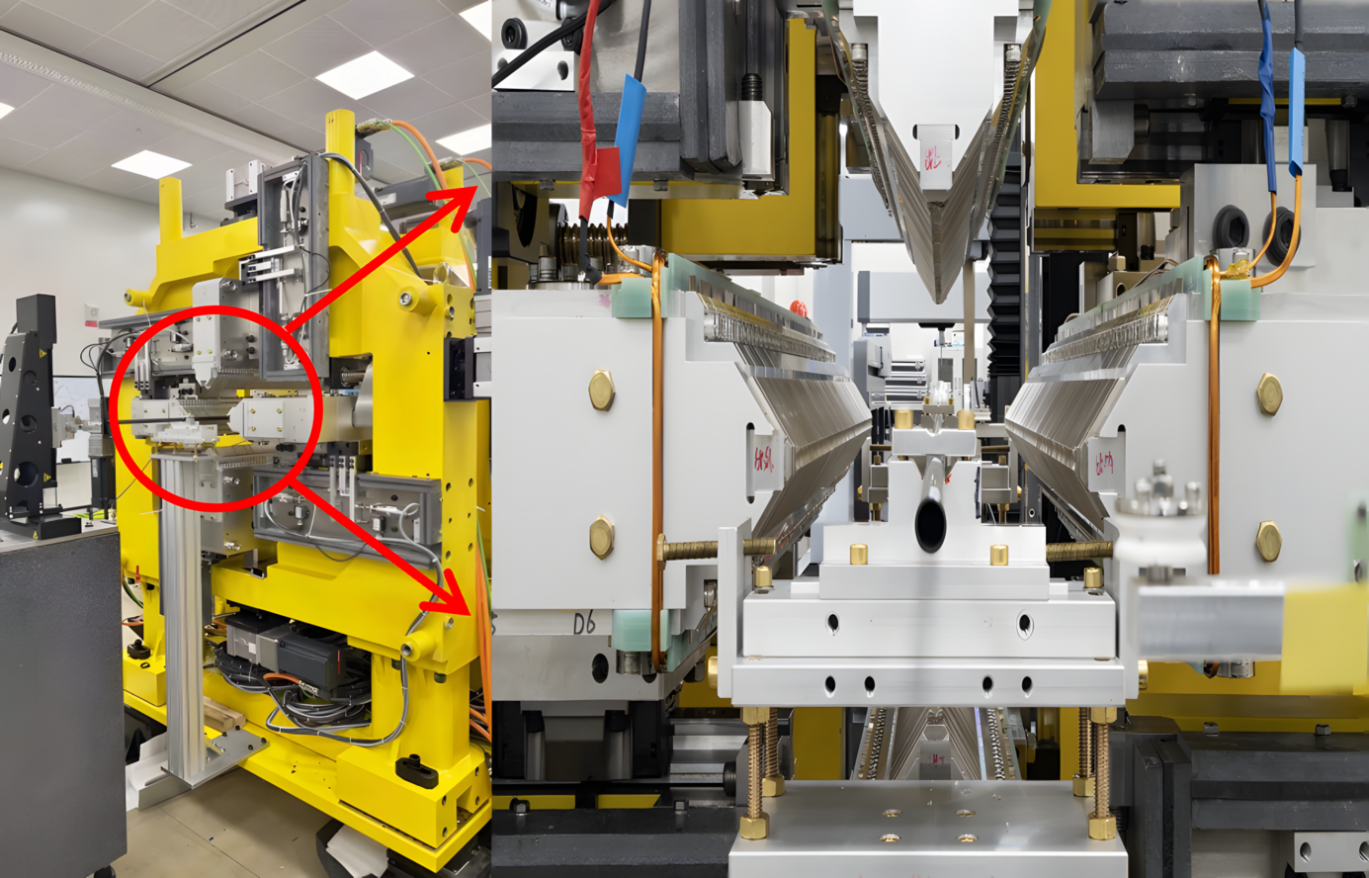
Photograph of the HEPS Mango wiggler.

**Figure 20:**
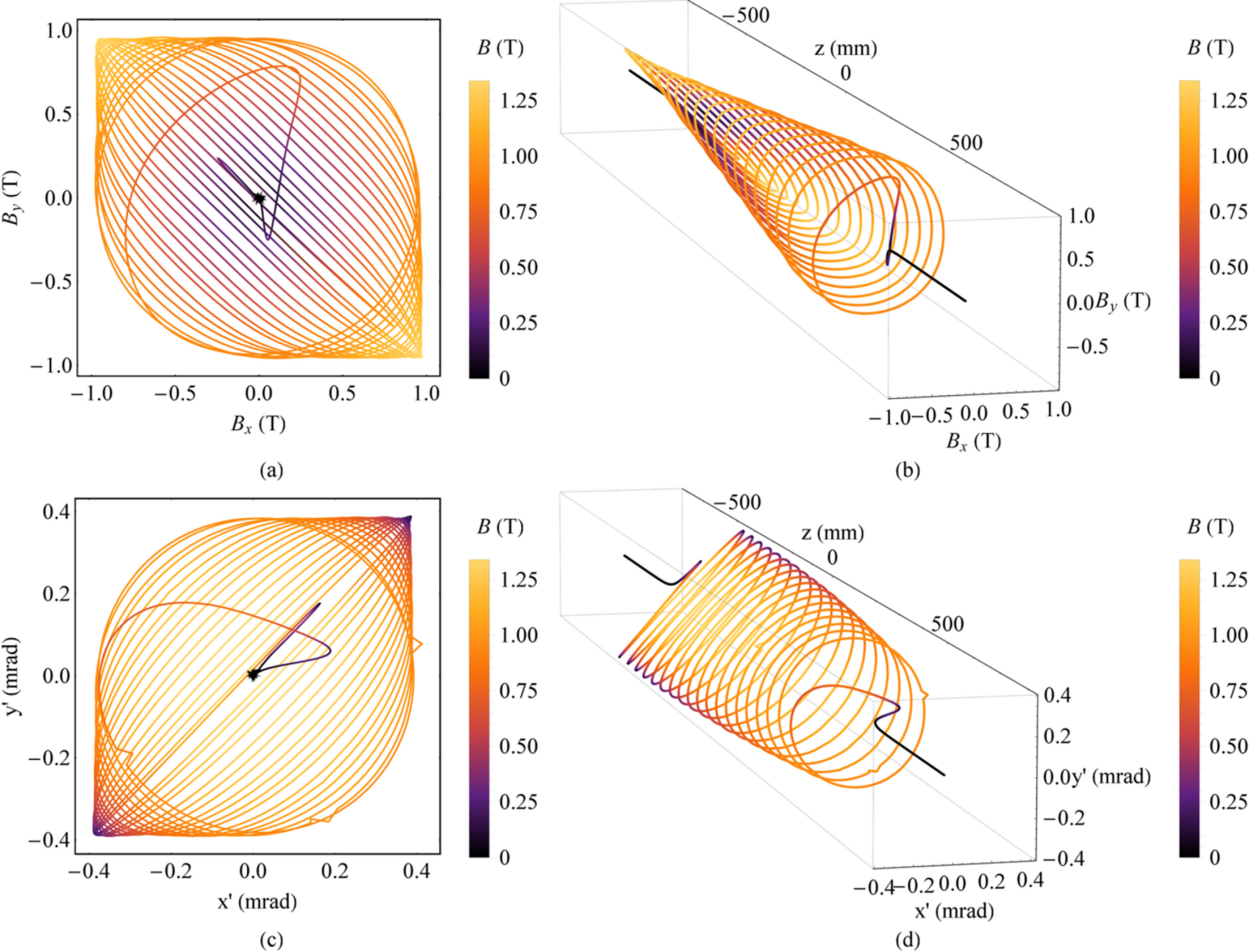
Magnetic field and angular trajectory of electrons in the HEPS Mango wiggler according to the data measured at the smallest gap (12.1 mm). (*a*) Magnetic field. (*b*) Magnetic field along *z*. (*c*) Angular trajectory of electrons. (*d*) Angular trajectory of electrons along *z*.

The HEPS is a fourth-generation SR source that is currently under construction. We developed a Mango wiggler for X-ray in-line imaging at HEPS. The parameters of the Mango wiggler at HEPS are listed in Table 1 ▸. The NdFeB magnet arrays of the delta type were adopted. This Mango wiggler works outside of vacuum. A practical Mango wiggler was constructed, as shown in Fig. 19 ▸, and measurements and adjustments of its magnetic field were carried out. The measured magnetic field and the trajectory at the smallest gap (12.1 mm) are shown in Fig. 20 ▸. The field errors and effects on the electron trajectory can be found in Fig. 20 ▸.

**Figure 21:**
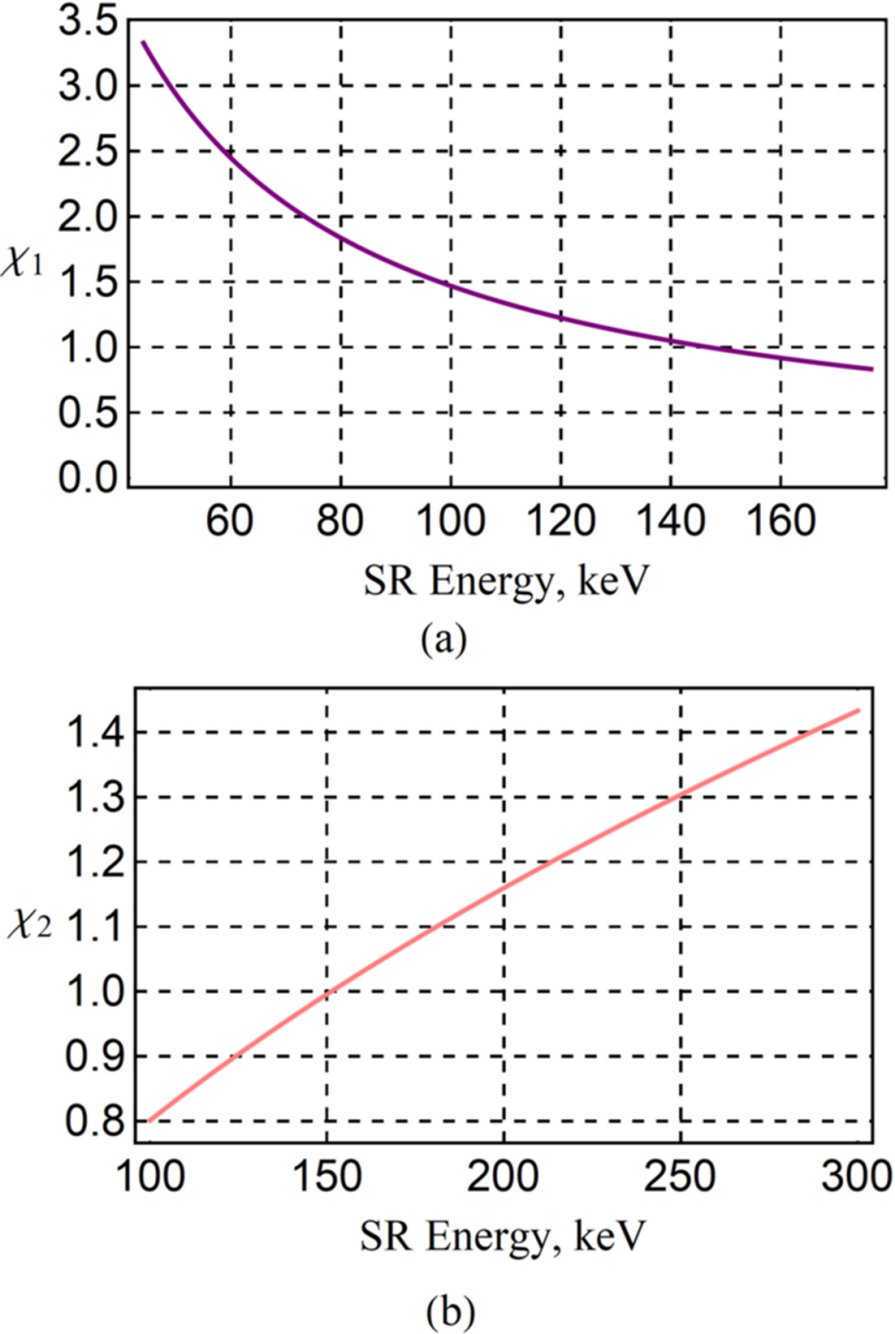
The designed parameters of the HEPS Mango wiggler and the parameters of the storage ring were substituted into equations (13) and (17). (*a*) χ_1_ with an energy dependence. (*b*) χ_2_ with an energy dependence.

**Figure 22:**
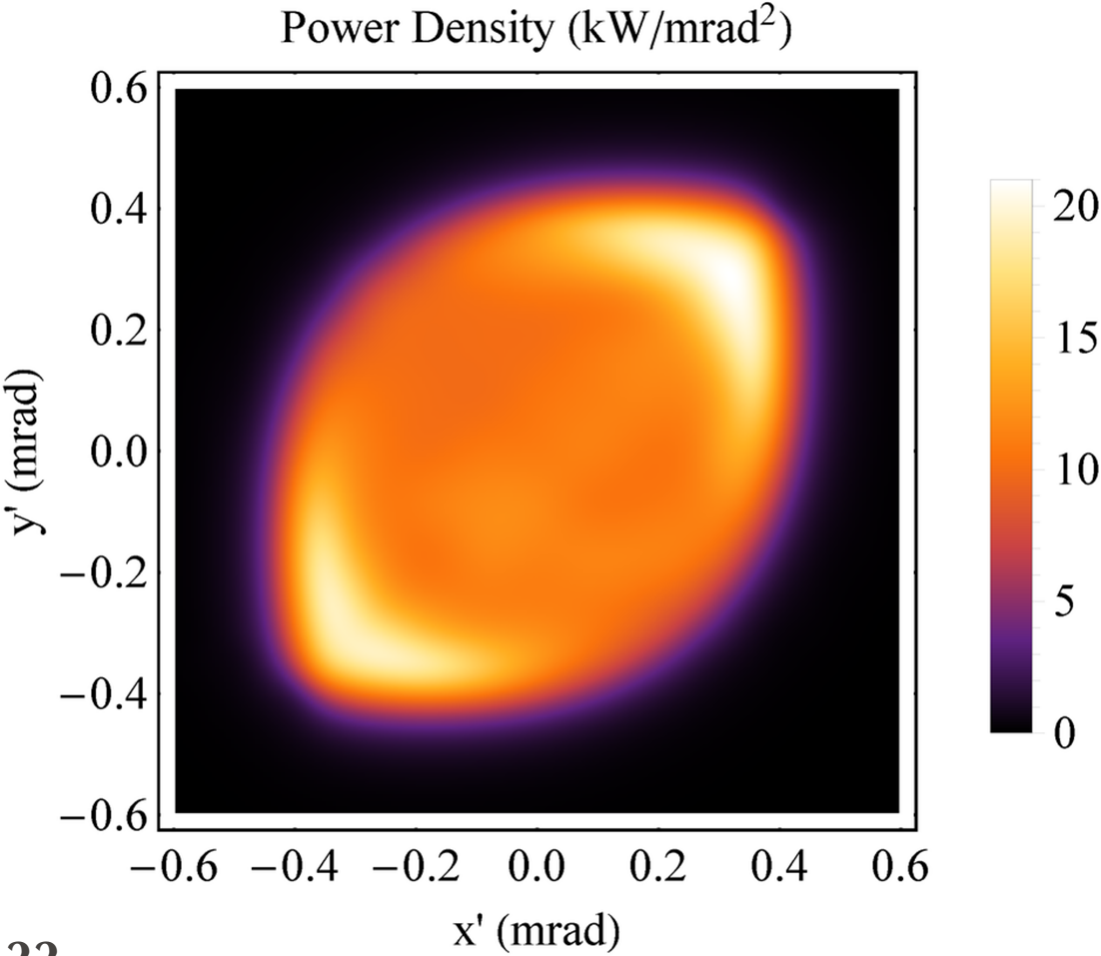
Simulated angular distribution of the power density of the HEPS Mango wiggler based on the magnetic field measured at the smallest gap (12.1 mm).

The energy of the electrons in the storage ring is 6 GeV; the divergences of the electron bunch σ_*x*′_ and σ_*y*′_ are 3.14 µrad and 1.20 µrad, respectively, in the middle of the insertion device. The energy spread δ_e_ is 0.0011. An SR energy range no higher than 300 keV is chosen. According to the analyses discussed in Section 3, the angular distribution of radiation is uneven when the SR energy is less than 48.8 keV (χ_1_ > 3); the angular distribution of radiation is very uniform from 147 keV to 151 keV (χ_1_ < 1 and χ_2_ < 1); and the angular distribution of radiation is in a transition state that can be used in many experiments from 48.8 keV to 147 keV (1 < χ_1_ < 3) and from 151 keV to 300 keV (1 < χ_2_ < 1.43) (see Fig. 21 ▸). Field measurements were carried out under a series of gaps. The data at the smallest gap (12.1 mm) were used to simulate the radiation at several SR energies (Figs. 22 ▸, 23 ▸, 24 ▸ and 25 ▸). The dark stripe in the centre of the FOV is caused by the longitudinal positioning deviation between the horizontal magnet rows and the vertical magnet rows.

## 5. Summary

A novel insertion device referred to as the Mango wiggler is introduced. It is constructed from two orthogonal planar wigglers with a small difference in their period lengths, eliciting the phase difference of the magnetic fields to incrementally transitions from 0 to π/2. The Mango wiggler can provide a large and symmetric FOV with a high flux and a high critical energy by expanding its vertical divergence to be the same as the horizontal divergence. As a result, the efficiency of SR X-ray in-line imaging experiments is improved while ensuring good imaging resolution. To study its radiation properties, detailed theoretical analyses and simulations were carried out via *SPECTRA* and *Mathematica*. The uneven angular distribution of radiation occurs at both low and high photon energies for different reasons. Mainly, the discretization of the spectrum results in the unevenness of the angular distribution of radiation at low energy. A tapered magnetic field can be applied to make the angular distribution of radiation at low energy more uniform. At high energy, a decrease in the SR divergence of the electron bunch results in longitude lines that cannot be uniformly filled. These theoretical analyses were then applied to the HEPS Mango wiggler and several simulations were conducted based on the measured magnetic field. Although the simulation results cannot provide final conclusions as the magnetic field requires further adjustment before the installation of the HEPS Mango wiggler, presenting the idea of the Mango wiggler is still meaningful.

## Figures and Tables

**Figure 7:**
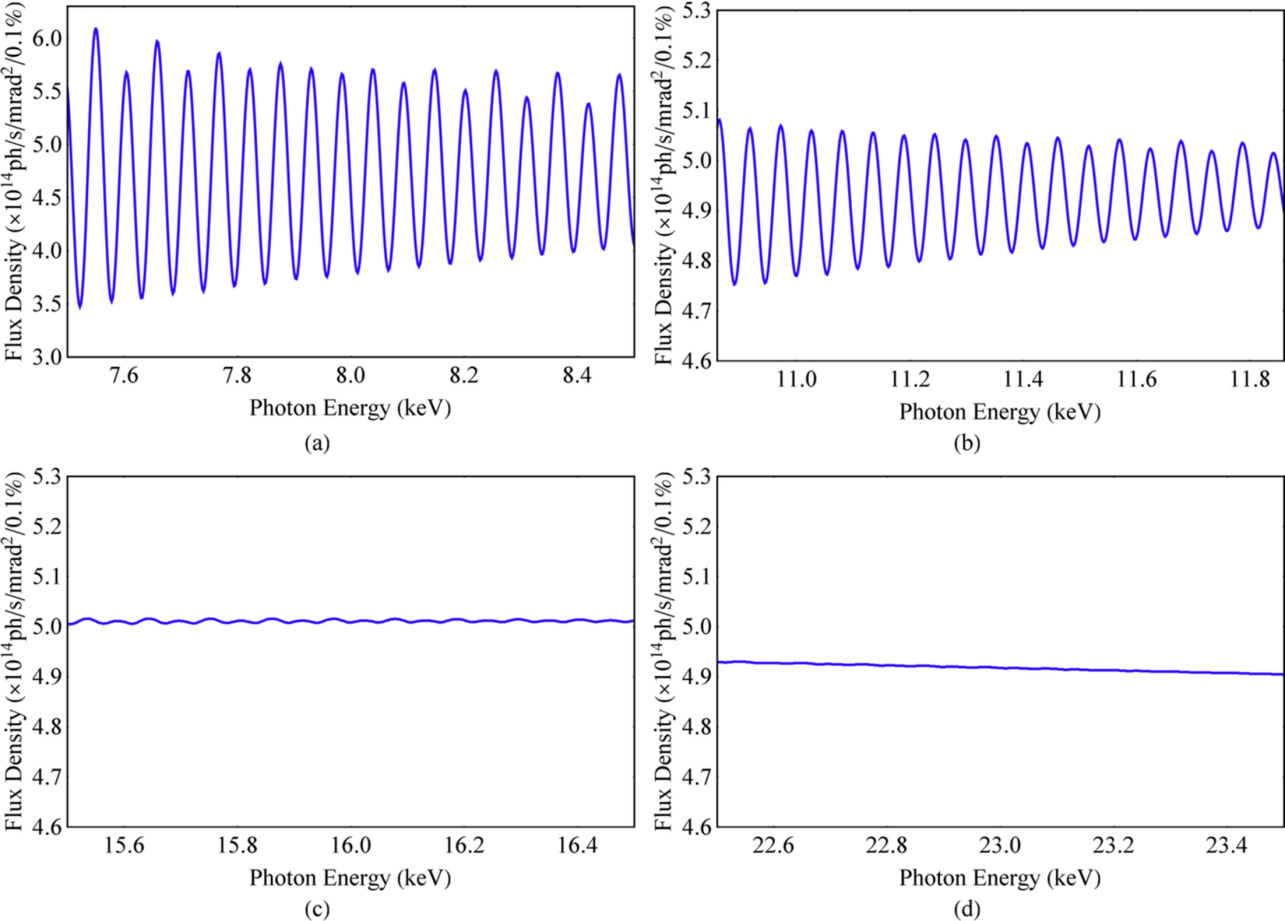
Flux density spectra of the ideal Mango wiggler along the beam axis: (*a*) SR energy around 8 keV, χ_1_ = 3.15. (*b*) SR energy around 11.36 keV, χ_1_ = 2.22. (*c*) SR energy around 16 keV, χ_1_ = 1.57. (*d*) SR energy around 23 keV, χ_1_ = 1.10.

**Figure 23:**
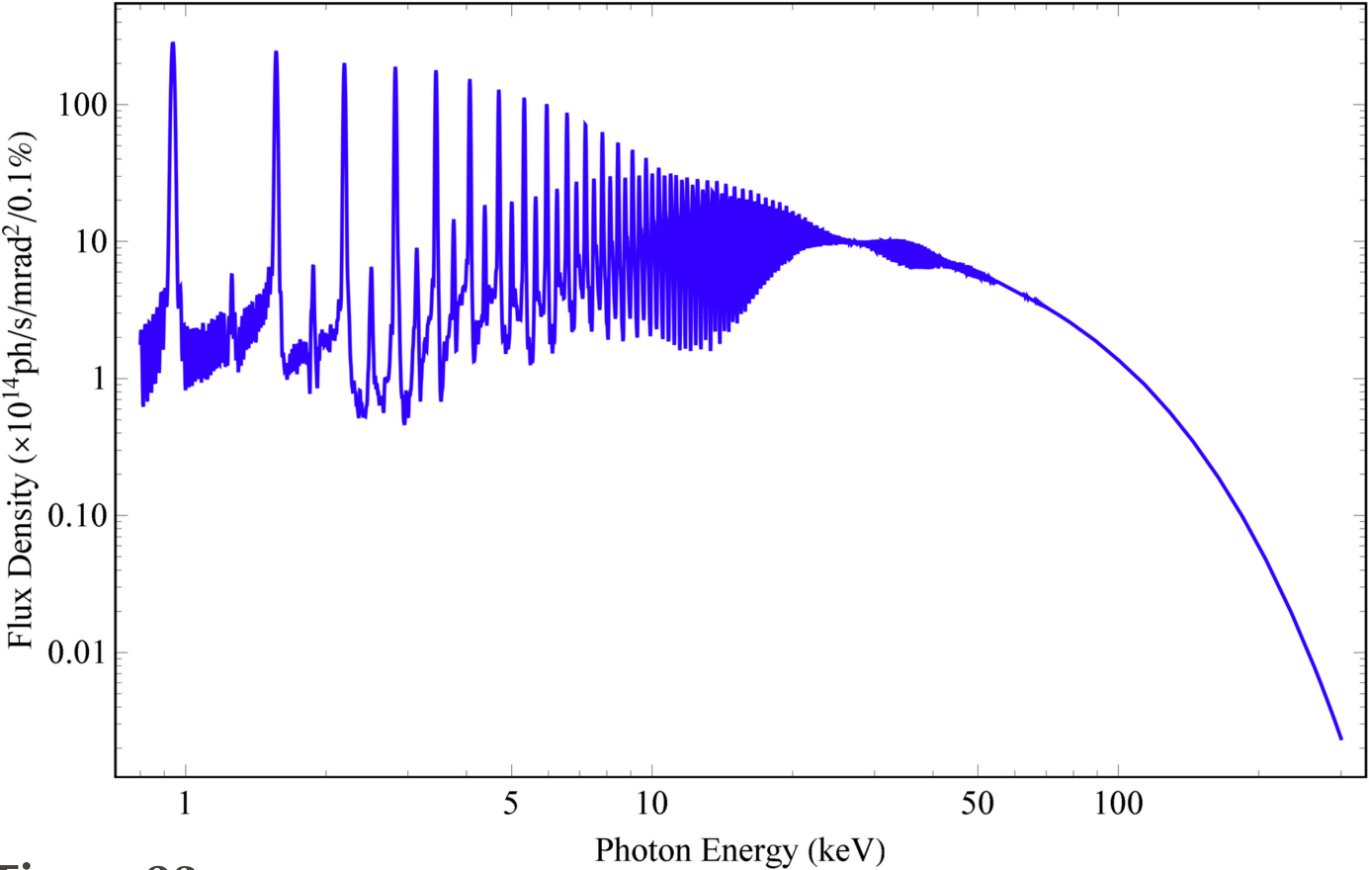
Flux density spectrum (0.8–300 keV, along the beam axis) of the HEPS Mango wiggler, simulated based on data measured at the smallest gap (12.1 mm).

**Figure 24:**
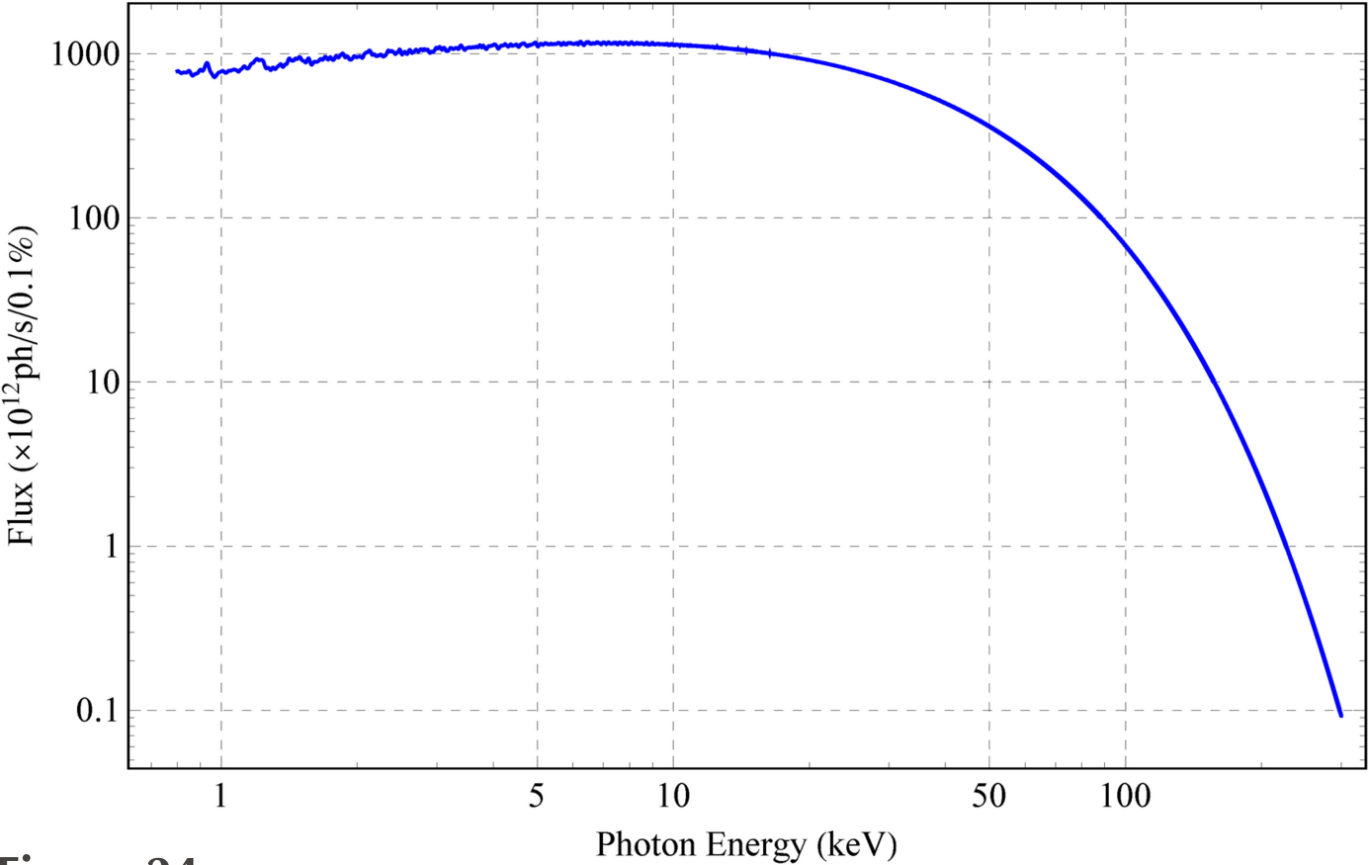
Photon flux spectrum (0.8–300 keV, within 1.5 mrad) of the HEPS Mango wiggler, simulated based on data measured at the smallest gap (12.1 mm).

**Figure 25:**
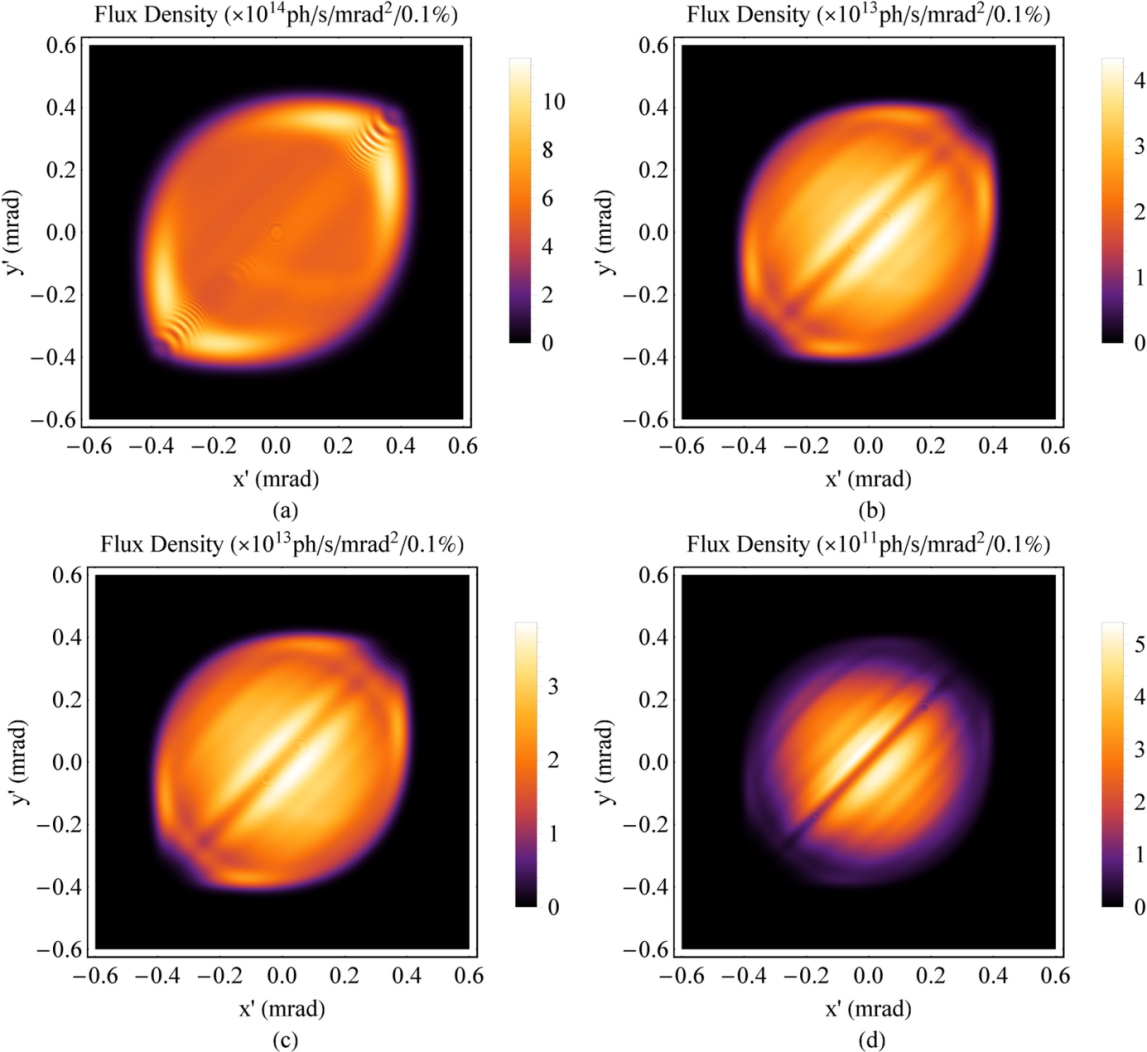
Angular distribution of the flux density for the HEPS Mango wiggler, simulated based on data measured at the smallest gap (12.1 mm). (*a*) SR energy at 48.8 keV. (*b*) SR energy at 147 keV. (*c*) SR energy at 151 keV. (*d*) SR energy at 300 keV.

**Table 1:** Parameters of the Mango wiggler designed for HEPS

Peak magnetic field strength, *B*_0_	1.0 T (0.2–1.0 T adjustable for the tapered mode)
Period lengths, λ_*ux*_/λ_*uy*_	50.70 mm/50 mm
Period, *N*_*ux*_/*N*_*uy*_	17.75/18
